# BCL-2 and BCL-xL in Cancer: Regulation, Function, and Therapeutic Targeting

**DOI:** 10.3390/ijms27021123

**Published:** 2026-01-22

**Authors:** João P. N. Silva, Bárbara Pinto, Patrícia M. A. Silva, Hassan Bousbaa

**Affiliations:** 1 UNIPRO—Oral Pathology and Rehabilitation Research Unit, University Institute of Health Sciences (IUCS), Cooperativa de Ensino Superior Politécnico e Universitário (CESPU), 4585-116 Gandra, Portugal; joaosilva_06@hotmail.com (J.P.N.S.); barbara.fernandes.15@gmail.com (B.P.); 2 Department of Physiology and Biophysics, Institute of Biological Sciences, Federal University of Minas Gerais (UFMG), Belo Horizonte 31270-901, Brazil; 3 Associate Laboratory i4HB—Institute for Health and Bioeconomy, University Institute of Health Sciences—CESPU, 4585-116 Gandra, Portugal; 4 UCIBIO—Applied Molecular Biosciences Unit, Translational Toxicology Research Laboratory, University Institute of Health Sciences (1H-TOXRUN, IUCS-CESPU), 4585-116 Gandra, Portugal

**Keywords:** BCL-2, BCL-xL, apoptosis regulation, cancer progression, drug resistance, therapeutic targeting, BH3 mimetics

## Abstract

The BCL-2 family of proteins plays a central role in the regulation of apoptosis, with BCL-2 and BCL-xL representing two of its most prominent antiapoptotic members. This review explores the molecular regulation of BCL-2 and BCL-xL genes, emphasizing the structural domains that define the functions of the broader BCL-2 family. Beyond their canonical roles in preventing mitochondrial outer membrane permeabilization, both proteins contribute significantly to cancer development. Their overexpression enhances invasiveness and tumor progression, supports angiogenesis, and critically modulates cellular responses to chemotherapy, often conferring drug resistance. Additional non-apoptotic functions, including roles in metabolism, mitochondrial dynamics, and cellular homeostasis, further expand their biological relevance. Clinical trials exploring strategies to inhibit BCL-2 and BCL-xL, including selective BH3 mimetics and combination regimens, are discussed with emphasis on their potential and limitations in oncology. Overall, this review highlights the multifaceted contributions of BCL-2 and BCL-xL to cancer biology and underscores the importance of continued efforts to refine targeted therapeutic approaches.

## 1. Introduction

**Figure 1:**
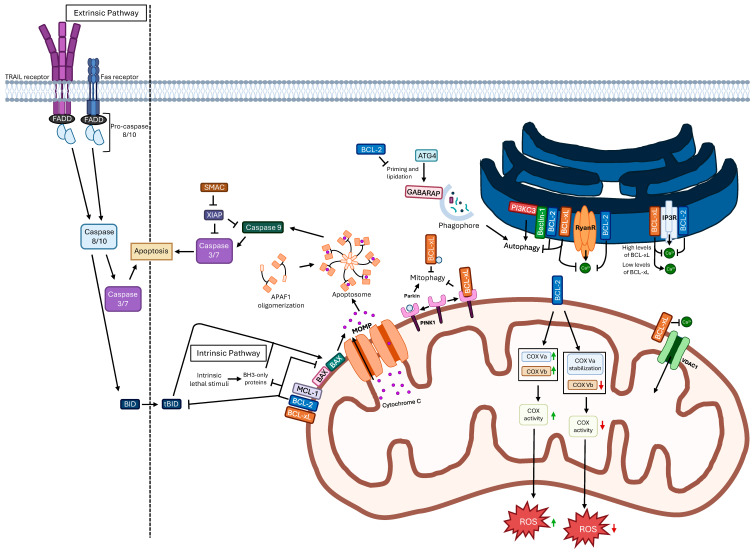
Extrinsic and intrinsic apoptotic pathways and BCL-2 and BCL-xL functions. Activation of the extrinsic pathway is initiated by extracellular death signals that activate death receptors, such as Fas and TRAIL receptors, which in turn activate caspase-8 and caspase-10 resulting in apoptosis induction. The intrinsic pathway is promoted by intracellular stress signals, including DNA damage, leading to repression of BCL-2 antiapoptotic protein members and activation of BH3-only proteins. This will lead to activation of pro-apoptotic proteins such as BAX and BAK, inducing MOMP that promotes caspases-3 and -7 activity resulting in apoptosis. BCL-2 is also involved in the regulation of ROS production by, in normal conditions, increasing ROS through the promotion of COX activity (green arrows), while under oxidative stress it decreases COX activity and consequently ROS levels (red arrows) and in autophagy repression by interfering with GABARAP lipidation. Both BCL-xL and BCL-2 prevent autophagy by inhibiting Beclin-1, and they can also inhibit ryanodine receptors and IP3 receptors preventing Ca^2+^ pro-apoptotic release. However, when in low levels, BCL-xL can promote Ca^2+^ release through IP3 receptors. BCL-xL can also inhibit VDAC1, preventing Ca^2+^ pro-apoptotic signaling from entering the mitochondria. The binding of BCL-xL to PINK1 and/or PARK2 inhibits mitophagy induction. Abbreviations: APAF-1, apoptotic protease activating factor 1; ATG4, autophagy-related 4; BAK, BCL-2 homologous antagonist/killer; BAX, BCL-2-associated X protein; BCL, B-cell lymphoma; BID, BH3-interacting domain death agonist; COX, cytochrome c oxidase; FADD, Fas-associated protein with death domain; GABARAP, GABA type A receptor-associated protein; IP3R, inositol 1,4,5-trisphosphate receptor; MCL-1, myeloid cell leukemia 1; MOMP, mitochondrial outer membrane permeabilization; PI3KC3, class III phosphatidylinositol 3-kinase complex; PINK1, PTEN-induced kinase 1; ROS, reactive oxygen species; RyanR, ryanodine receptor; SMAC, second mitochondria-derived activator of caspases; TRAIL, TNF-related apoptosis-inducing ligand; VDAC1, voltage-dependent anion channel 1; XIAP, X-linked inhibitor of apoptosis protein.

B-cell lymphoma (BCL)-2 and BCL-xL are two antiapoptotic members of the BCL-2 family that play central roles in regulating the intrinsic apoptotic pathway. Apoptosis is the most common type of programmed cell death in human tissues and, together with cell proliferation, contributes to tissue homeostasis, development, and remodeling, such as during the formation of the aortic arch [1,2]. There are two major pathways mediating apoptosis: the extrinsic and intrinsic pathways (Figure 1) [3].

The extrinsic pathway is initiated by extracellular death signals. Cell membranes express death receptors, such as Fas, TRAIL-R1/2, and TNF-R, that, upon binding to their respective ligands, recruit adaptor proteins like FADD to form the death-inducing signaling complex (DISC), resulting in the activation of initiator caspase-8 and caspase-10 [3,4]. In contrast, the intrinsic pathway is triggered by intracellular stress signals, including DNA damage, oncogenic activation, oxidative stress, or endoplasmic reticulum (ER) stress, which converge on the mitochondria and lead to mitochondrial outer membrane permeabilization (MOMP) [3]. Members of the BCL-2 family tightly regulate this pathway [5]. These proteins are organized into three major functional groups: (i) antiapoptotic proteins (BCL-2, BCL-xL, myeloid cell leukemia-1 (MCL-1), BCL-w, BCL-2-related gene A1 (A1/BFL-1), and BCL-B), (ii) pro-apoptotic effectors (BCL-2-associated X protein (BAX), BCL-2 homologous antagonist/killer (BAK), and BCL-2-related ovarian killer (BOK)), and (iii) BH3-only activators and sensitizers (such as BCL-2-interacting mediator of cell death (BIM), BH3-interacting domain death agonist (BID), p53 upregulated modulator of apoptosis (PUMA), NOXA, BCL-2-modifying factor (BMF), BCL-2 interacting killer (BIK), BCL-2 agonistic of cell death (BAD), and Harakiri (HRK)). Their interactions are mediated through conserved BCL-2 homology (BH) domains, which allow antiapoptotic proteins to sequester BH3-only factors or directly inhibit BAX/BAK oligomerization, thereby preventing MOMP. For instance, BIK binds and inhibits pro-survival proteins, including BCL-xL, thereby promoting MOMP; given BIK’s ER localization, the interaction is thought to occur at ER–mitochondria contact sites [6]. MOMP results in the release of pro-apoptotic factors, most notably cytochrome c, from the mitochondrial intermembrane space into the cytosol, where cytochrome c associates with APAF-1 and procaspase-9 to form the apoptosome [7]. This complex activates caspase-9 (intrinsic pathway), or, in the extrinsic route, caspase-8 and caspase-10 directly activate downstream effectors. Ultimately, both pathways converge on the activation of executioner caspases, such as caspase-3 and caspase-7, which cleave essential structural and regulatory proteins, leading to the morphological and biochemical hallmarks of apoptotic cell death [8]. While the BCL-2 family comprises several proteins with pro- or antiapoptotic functions, BCL-2 and BCL-xL have been particularly implicated in tumor cell survival and therapeutic response pathways in cancer [9]. Both BCL-2 and BCL-xL are among the most studied antiapoptotic regulators of the intrinsic apoptotic pathway, acting as central control points for MOMP, the critical step that determines whether a cell will commit to apoptosis [10]. MOMP is tightly coupled to mitochondrial metabolic status. Mitochondria integrate bioenergetic flux, redox balance, and calcium signaling, all of which critically influence apoptotic susceptibility. Antiapoptotic proteins such as BCL-2 and BCL-xL not only prevent MOMP by inhibiting BAX/BAK activation but also modulate mitochondrial metabolism by regulating oxidative phosphorylation efficiency, mitochondrial membrane potential, and ATP production [11,12]. In particular, BCL-xL has been shown to interact with components of the F_1_F_0_-ATP synthase, enhancing mitochondrial energetic efficiency and promoting cell survival under metabolic stress [12]. Moreover, BCL-2 family members localize to mitochondria-associated membranes, where they regulate ER–mitochondria calcium transfer, thereby linking metabolic signaling to apoptotic priming [13]. Metabolic stress conditions, such as nutrient deprivation or altered mitochondrial respiration, can sensitize cells to apoptosis by shifting the balance of BCL-2 family interactions, influencing cytochrome c release and caspase activation [14]. These metabolic–apoptotic interconnections are particularly relevant in cancer cells, where metabolic reprogramming contributes to apoptotic resistance and impacts the therapeutic efficacy of BCL-2 and BCL-xL inhibitors [14].

Because apoptosis is a fundamental tumor-suppressive mechanism, its evasion is considered a hallmark of cancer. BCL-2 and BCL-xL are frequently dysregulated across malignancies, where their overexpression supports tumor cell survival, enhances chemoresistance, and facilitates disease progression [15]. Functional assays such as BH3 profiling have shown that cancers can exhibit a specific dependency on BCL-2 or BCL-xL for survival, reflecting a measurable shift in apoptotic threshold that correlates with therapeutic sensitivity or resistance in different tumor types, a pattern less consistently observed for other antiapoptotic family members [16,17]. This dependency is not uniform across all antiapoptotic family members but is especially evident for BCL-2 and BCL-xL in various hematologic and solid tumor contexts, where their relative expression and binding affinities dictate apoptotic control and drug responsiveness [18].

Beyond apoptosis suppression, these proteins also influence metabolic adaptation, mitochondrial dynamics, calcium signaling, angiogenesis, and cell migration, highlighting their multifaceted roles in tumor biology [19]. Clinically, the therapeutic relevance of the BCL-2 family is underscored by the success of BH3 mimetics such as Venetoclax, approved for hematologic malignancies, although resistance mechanisms, particularly involving MCL-1 and BCL-xL, remain important challenges. In this review, we summarize the structural and functional characteristics of BCL-2 and BCL-xL, highlight their contribution to tumor biology, and discuss clinical trials exploring current therapeutic strategies targeting these key survival proteins.

## 2. BCL-2 and BCL-xL Genes and Regulation

**Figure 2:**
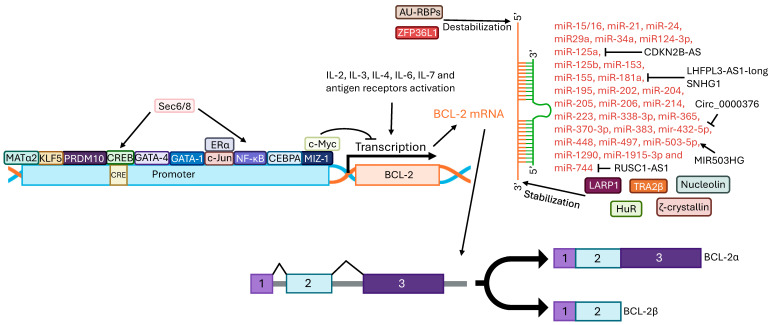
Representation of the regulation of BCL-2 at the transcriptional and post-transcriptional levels. Several transcriptional factors can regulate *BCL2* expression by binding to its promoter. For instance, PRDM10, CREB (which binds to the CRE region in the promoter), GATA-4, GATA-1, c-Jun associated with ERα, NF-κB, CEBPA, and MIZ-1 were found to bind the promoter and contribute to *BCL2* transcription. IL-2, IL-3, IL-4, IL-6, IL-7, Sec6, Sec8, and antigen receptor activation also promote *BCL2* expression. On the other hand, c-Myc was found to bind to MIZ-1 to repress *BCL2* transcription. At the post-transcriptional level, several miRNAs have been found to bind and repress BCL-2 translation. Further, AU-RBPs and ZFP36L1 can bind BCL-2 mRNA to destabilize it. Conversely, LARP1, TRA2β, Nucleolin, HuR, and ζ-crystallin promote its stabilization. LncRNAs and circular RNAs can act as CeRNAs and regulate microRNAs activity. Two isoforms of BCL-2 exist: a longer one known as BCL-2α and defined by its antiapoptotic activity and a shorter one with no known activity. Abbreviations: AU-RBPs, adenylate-uridylate-rich element RNA-binding protein 1; BCL-2, B-cell lymphoma-2; CEBPA, CCAAT enhancer-binding protein alpha; CRE, cAMP response element; CREB, cAMP response element-binding protein; ERα, estrogen receptor α; HuR, human antigen R; IL, interleukin; KLF5, Krüppel-like factor 5; LARP1, La-related protein 1; MATα2, methionine adenosyltransferase α2; miR, microRNA; MIZ-1, Myc-interacting zinc finger protein-1; mRNA, messenger ribonucleic acid; NF-κB, nuclear factor κB; PRDM10, PR/SET domain 10; SNHG1, small nucleolar RNA host gene 1; TRA2β, transformer 2β homolog; ZFP36L1, zinc finger protein 36, C3H type-like 1.

The *BCL2* gene is located on chromosome 18 (18q21.33) and is constituted by three exons (Figure 2). The BH domains are encoded by the first two exons, while the third encodes the transmembrane domain [20,21].

Transcription of *BCL2* is promoted in response to signaling from diverse cytokines, such as interleukin (IL)-2, IL-3, IL-4, IL-6 and IL-7, or upon activation of antigen receptors which leads to the stimulation of several transcription factors [22]. In the endometrium and glandular cells, *BCL2* gene transcription seems to be regulated by c-Jun. It is possible that, in the endometrium, this regulation depends on the interaction of c-Jun with estrogen–estrogen receptor α [23]. PR/SET domain 10, an epigenetic regulator involved in development and cell differentiation, also promotes BCL-2 mRNA expression by binding to *BCL2* gene promoter [24]. Furthermore, the transcription factor GATA-1 is also involved in *BCL2* transcription regulation, while GATA-4 was found to promote BCL-2 expression in both normal ovarian cells and ovarian granulosa cell tumors [25,26]. Moreover, NF-κB overexpression was associated with a six-fold increase in *BCL2* promoter transcriptional activity, whereas mutations in the promoter eliminated this effect [27]. The *BCL2* promoter also presents a CRE site where the transcriptional factor CREB, when phosphorylated, can bind and promote BCL-2 expression [28]. Sec6 and Sec8 were found to regulate *BCL2* transcription by modulating CREB and NF-κB activity in malignant peripheral nerve sheath tumor cells. Depletion of Sec6 and Sec8 decreases BCL-2 expression [29].

In colon and liver cancer, methionine adenosyltransferase α2 was found to bind to the *BCL2* promoter and induce its expression, but also to the BCL-2 protein stabilizing it [30].

On the other hand, c-Myc can repress BCL-2 expression to promote apoptosis. This regulation appears to require c-Myc binding to the transcription factor MIZ-1, leading to its functional inactivation [31].

At the post-transcriptional level several proteins have been reported to promote stability or to destabilize BCL-2 mRNA. In glioblastoma, human antigen R was found to bind to the 3′ untranslated region of both BCL-2 and BCL-xL mRNAs, stabilizing them and preventing their degradation [32]. Similarly, nucleolin has also been shown to bind to 3′ untranslated region in BCL-2 and BCL-xL mRNAs, stabilizing them [33]. Furthermore, ζ-crystallin, transformer 2β (TRA2β), and La-related protein 1 also act as stabilizers of BCL-2 in different types of cancer [34,35,36]. On the other hand, adenylate-uridylate-rich element RNA-binding protein 1 and zinc finger protein 36, C3H1 type-like 1 have been found to destabilize BCL-2 mRNA, leading to its degradation and consequently decreasing BCL-2 protein levels [35,37,38]. The following miRNAs have also been reported to regulate BCL-2 mRNA levels: miR-15/16, miR-21, miR-24, miR-29a, miR-34a, miR-124-3p, miR-125a, miR-125b, miR-153, miR-155, miR-181a, miR-195, miR-202, miR-204, miR-205, miR-206, miR-214, miR-223, miR-338-3p, miR-365, miR-370-3p, miR-383, miR-432-5p, miR-448, miR-497, miR-503-5p, miR-744, miR-1290, and miR-1915-3p [39,40,41,42,43,44,45,46,47,48,49,50,51,52,53,54].

Moreover, BCL-2 has two protein isoforms: BCL-2α, an antiapoptotic isoform with 239 amino acids, and BCL-2β, with 205 amino acids and no known function [21].

**Figure 3:**
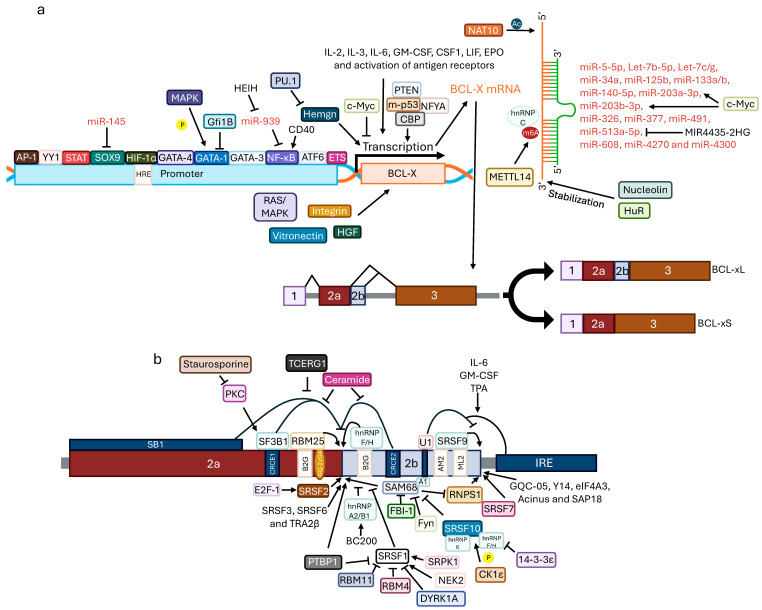
Representation of the regulation of BCL-xL at the transcriptional and post-transcriptional levels. (**a**) Several transcriptional factors can regulate *BCL-X* expression by binding to its promoter. For instance, AP-1, YY1, HIF-1α (which binds to the HRE region in the promoter), STAT, GATA-4, GATA-1, GATA-3, SOX9, NF-κB, ATF6, and ETS were found to bind the promoter and contribute to *BCL-X* transcription. MAPK phosphorylates GATA-1, enhancing its activity while Gli-1B antagonizes GATA-1. IL-2, IL-3, IL-6, GM-CSF, CSF1, LIF, Integrin, HGF, Vitronectin, EPO, Hemgn, and antigen receptor activation also promote *BCL-X* expression. When p53 is mutated PTEN can form a complex with it, NFYA and acetyl-CBP to induce *BCL-X* transcription. On the other hand, c-Myc can repress *BCL-X* transcription. At the post-transcriptional level, several miRNAs have been found to bind and repress BCL-xL translation. However, Nucleolin, HuR, and acetylation mediated by NAT10 promote *BCL-X* mRNA stabilization. LncRNAs can act as CeRNAs and regulate microRNAs activity and also repress transcriptional factors expression. METTL14 can also increase the m6A level at the A1001 site of the *BCL-X* mRNA recruiting hnRNP C to promote BCL-xL expression. (**b**) Several proteins are involved in the splicing regulation of *BCL-X* pre-mRNA promoting the expression of BCL-xL or BCL-xS. Abbreviations: Ac, acetyl; AP-1, activator protein 1; ATF6, activating transcription factor 6; BC200, brain cytoplasmic 200; BCL, B-cell lymphoma; CBP, CREB binding protein; CK1ε, casein kinase 1ε; CSF1, colony-stimulating factor-1; DYRK1A, dual-specificity tyrosine-phosphorylation-regulated kinase 1A; eIF4A3, eukaryotic initiation factor 4A-III; EPO, erythropoietin; ETS, E26 transformation-specific; FBI-1, factor binding to inducer of short transcripts-1; Gfi1B, growth factor independence 1B; GM-CSF, granulocyte-macrophage colony-stimulating factor; HEIH, hepatocellular carcinoma up-regulated EZH2-associated lncRNA; HGF, hepatocyte growth factor; HIF-1α, hypoxia-inducible factor; hnRNP, heterogeneous nuclear ribonucleoprotein; HRE, hypoxia-responsive element; HuR, human antigen R; IL, interleukin; IRE, iron-responsive element; LIF, leukemia inhibitory factor; MAPK, mitogen-activated protein kinase; METTL14, methyltransferase-like 14; miR, microRNA; m-p53, mutant-p53; mRNA, messenger ribonucleic acid; NAT10, *N*-acetyltransferase 10; NEK2, never in mitosis A-related kinase 2; NF-κB, nuclear factor κB; NFYA, nuclear transcription factor Y α; PKC, protein kinase C; PTBP1, polypyrimidine tract binding protein 1; PTEN, phosphatase and tensin homolog; RBM, RNA binding motif protein; RNPS1, RNA-binding protein with SR domain 1; SAM68, SRC associated in mitosis, 68 kDa; SAP18, Sin3A-associated protein, 18kDa; SF3B1, splicing factor 3b subunit 1; SOX9, SRY-box transcription factor 9; SRPK1, serine/arginine-rich protein-specific kinase 1; SRSF, serine/arginine splicing factor; STAT, signal transducer and activator of transcription; TCERG1, transcription elongation regulator 1; TPA, 12-O-tetradecanoylphorbol-13-acetate; TRAF2β, tumor necrosis factor receptor-associated factor 2β; YY1, Yin Yang 1; U1, U1 small nuclear ribonucleoprotein.

The *BCL-X* gene, also known as *BCL2L1*, localizes on chromosome 20 (20q11.21) [55]. The expression of *BCL-X* is induced by IL-2, IL-3, IL-6, granulocyte-macrophage colony-stimulating factor, colony-stimulating factor-1, leukemia inhibitory factor, erythropoietin, and also by the activation of antigen receptors (Figure 3) [22]. The *BCL-X* gene transcription is regulated by several transcription factor families such as STAT, NF-κB, E26 transformation specific sequence, and activator protein 1 complex [56]. Its expression is also stimulated by integrin, vitronectin, hepatocyte growth factor, and the activated RAS/mitogen-activated protein kinase (MAPK) pathway [22]. In prostate cancer cells, hypoxia-inducible factor (HIF)-1α was found to directly bind to a region of *BCL-X* promoter known as hypoxia-responsive element promoting BCL-xL transcription [57]. Furthermore, type 2 calreticulin mutations in myeloproliferative neoplasms were associated with activation of the transcriptional factor activating transcription factor 6, leading to increased transcription of BCL-xL [58]. Upon CD40 stimulation the NF-κB subunits p65 and p52 in chronic lymphocytic leukemia (CLL) cells bind to the *BCL-X* promoter, inducing BCL-xL expression [59]. MAPK-mediated phosphorylation of GATA-1 can also promote BCL-xL transcription. Nonetheless, GATA-1 activity is antagonized by Gfi1B [26].

GATA-3 and GATA-4 have also been reported to regulate transcription of BCL-xL [26]. Nonetheless, BCL-xL expression can be repressed by c-Myc to promote apoptosis [31].

Due to alternative splicing two major mRNA isoforms exist: BCL-xL, with 780 base pairs, and BCL-xS, with 591 base pairs [55]. The splicing is highly regulated involving several proteins like SRC associated with mitosis, of 68 kDa (SAM68), alternative splicing factor (ASF) 1/Serine/arginine-rich splicing factor (SRSF) 1, heterogeneous nuclear ribonucleoproteins (hnRNPs), RNA binding motif protein (RBM) 25, and RBM4 [56,60].

For instance, when SAM68 is overexpressed and associated with hnRNP A1, which is regulated by Fyn kinase, it promotes BCL-xS production, while when depleted BCL-xL production is induced [60]. However, factor that binds to inducer of short transcripts 1, a transcription factor, SRSF1 and SRSF10 can inhibit SAM68, promoting BCL-xL expression [60,61]. In lung cancer, the serine/threonine kinase CK1ε was found to phosphorylate SRSF10, which is suggested to be necessary for SRSF10 to bind to *BCL-X* pre-mRNA, leading to BCL-xL expression [62]. Nonetheless, SRSF10 can also promote BCL-xS in association with SAM68 and hnRNP A1/A2 in response to DNA damage. SRSF1 is positively regulated by NEK2 and SR protein kinase 1, but negatively regulated by PTBP1, also known as hnRNP I, and RBM4, which compete for the same binding sites [63]. Moreover, other proteins that can bind to RNA G-quadruplexes, usually found in introns, and G-quadruplex stabilizing small molecules, such as GQC-05, can also modulate *BCL-X* splicing. For instance, GQC-05 was shown to shift splicing to express BCL-xS [64]. U1 snRNP, hnRNP F, hnRNP H, RBM25, RBM10, RBM4, SRSF2, SRSF3, TRA2β, and staurosporine also promote BCL-xS expression [60,63,65,66,67,68]. Nonetheless, in glioma cells, SRSF1 and SRSF2 were shown to promote BCL-xL expression, whereas SRSF6 favored BCL-xS expression. The truncated form of dual-specificity and tyrosine phosphorylation-regulated protein kinase 1A, involved in Alzheimer’s disease, however, inhibits SRSF1, enhancing production of the BCL-xS isoform [69]. Similarly, RBM11 also antagonizes SRSF1 [70]. Ceramide, a component lipid that regulates growth pathways and cell stress responses, has also been implicated in the splicing of *BCL-X* by promoting BCL-xS production [63].

On the other hand, hnRNP K, SRSF7, splicing-factor-3B-subunit-1 (SF3B1), and SRSF9 are involved in the expression of BCL-xL [60,66,71]. Core and auxiliary proteins, like Y14, eukaryotic translation initiation factor 4A-III, RNA-binding protein with SR domain 1 (RNPS1), acinus, and Sin3A-associated protein, 18 kDa, part of the exon junction complex, were also shown to promote BCL-xL production since their depletion shifted the alternative splicing in favor of BCL-xS [72]. In addition, the SB1 region in the *BCL-X* pre-mRNA can be bound by a repressor of BCL-xS isoform. A slower RNA polymerase II (RNAPII) rate of elongation can allow the repressor to bind to this region and induce BCL-xL production. However, the splicing-related factor transcription elongation regulator 1 was shown to modulate the RNAPII rate of elongation shifting the production of BCL-xL to BCL-xS [73]. In multiple myeloma, *N*-acetyltransferase 10 was found to acetylate BCL-xL mRNA, stabilizing it and allowing upregulation of BCL-xL which is essential for the activation of the phosphatidylinositol 3-kinase (PI3K)/AKT pathway promoting cancer proliferation and progression [74].

At the post-transcriptional level, miRNAs regulate BCL-xL expression. For instance, the miRNAs miR-5-5p, Let-7b-5p, Let-7c/g, miR-34a, miR-125b, miR-133a/b, miR140-5p, miR-203a-3p, miR-203b-3p, miR-326, miR-377, miR-491, miR-608, miR-4270, and miR4300 all target BCL-xL [22,39,60,75,76,77]. In pancreatic cancer, the long non-coding RNA MIR4435-2HG was found to act as ceRNA that binds to miR-513a-5p, promoting *BCL-X* expression inducing cell proliferation [78].

Besides BCL-xL, antiapoptotic form with 233 amino acids, and BCL-xS, pro-apoptotic form with 170 amino acids, a third protein isoform, BCL-xβ, with 277 amino acids and no known function exists [55,56].

## 3. BCL-2 Family Members Domains and Functions

**Figure 4:**
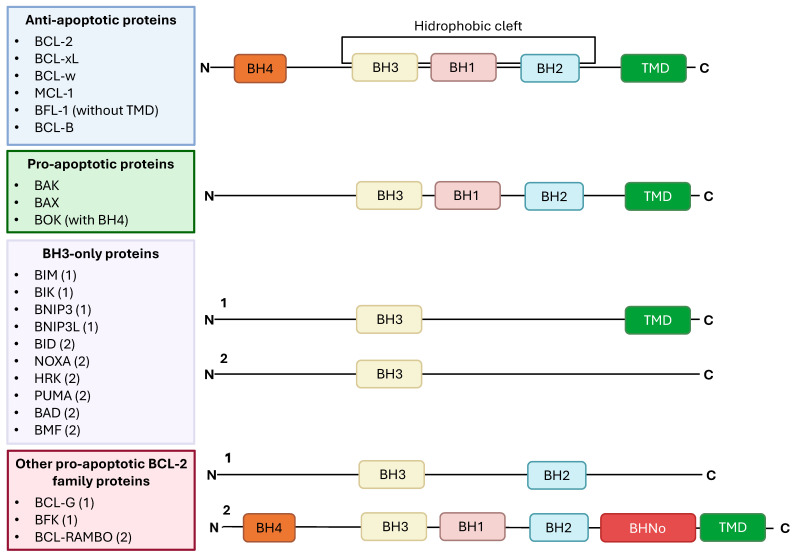
Schematic representation of the domains of BCL-2 family members. BCL2 family members can be divided into three categories: antiapoptotic, pro-apoptotic, and BH3-only proteins. The first comprises BCL-2, BCL-xL, BCL-w, MCL-1, BFL-1, and BCL-B. BFL-1 is the only member of this group which does not have the transmembrane domain. The second is constituted by BAK, BAX, and BOK. BOK is the only member with a BH4 domain. The third group can be divided into members that possess a transmembrane domain such as BIM, BIK, BNIP3, BNIP3L, and members that do not have a transmembrane domain like BID, NOXA, HRK, PUMA, BAD, and BMF. Other members of the BCL-2 family do not fit into these categories, such as BCL-G and BFK which only possess BH3 and BH2 domains. BCL-RAMBO is the only known member to have a BHNo domain.

BCL-2 family members can be divided in three categories: prosurvival, which include BCL-2 and BCL-xL, containing four BH domains (BH1 to BH4); pro-apoptotic, such as BAX and BAK, containing three BH domains (BH1 to BH3); and another category comprising proteins that only contain the BH3 domain, like BAD and BIM (Figure 4) [5,79,80]. However, several other BCL-2 family proteins, such as BCL-G, BCL-2 family kin (BFK), and BCL-RAMBO, do not fit in these categories.

Nonetheless, several members have a hydrophobic C-terminal region suggested to function as a transmembrane anchor [5,79].

The BH1–3 domains form a hydrophobic loop which is needed for BCL-2 and BCL-xL to interact with apoptotic regulators, including BH3-only proteins, and perform their antiapoptotic activity [80].

The BH4 domain is essential for several functions of BCL-2 and BCL-xL. For instance, this domain is necessary for the heterodimerization of BCL-2 and BAX which leads to BAX inhibition [81]. Furthermore, phosphorylation of BCL-2 at serine (Ser) 87 is important for this interaction since it decreases BCL-2’s affinity to BAX [82].

**Figure 5:**
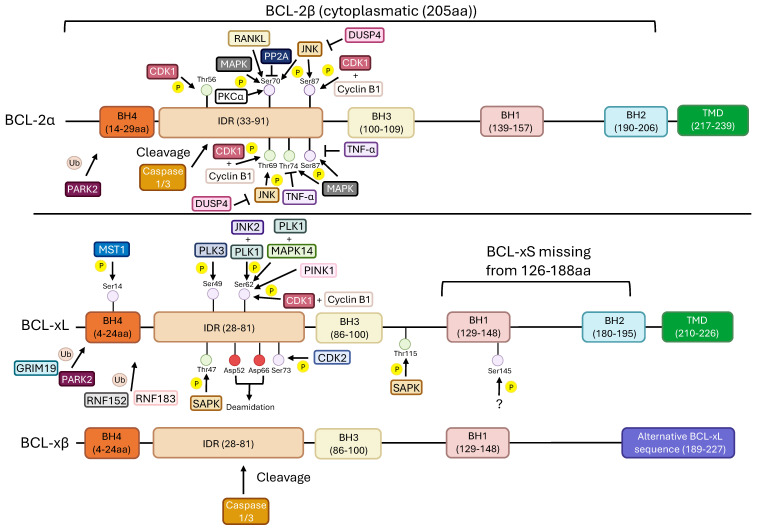
BCL-2 and BCL-xL post-translational regulation. Both proteins are highly regulated mostly through the phosphorylation of several serine and threonine residues regulating their activity. Ubiquitination (Ub) of both proteins has also been reported and leads to their degradation, however PARK2-mediated mono-ubiquitination of BCL-2 seems to promote the interaction between BCL-2 and Beclin-1. Caspase-1 and -3 can cleave the IDR regions of BCL-2 and BCL-xL, shifting their activity from antiapoptotic to pro-apoptotic. ? refers to unknown proteins. Abbreviations: Asp, asparagine; BCL, B-cell lymphoma; BH, BCL-2 homology; CDK, cyclin-dependent kinase; DUSP4, dual specificity phosphatase 4; GRIM19, gene associated with retinoid-interferon-induced mortality-19; IDR, intrinsically disordered region; JNK, c-Jun *N*-terminal kinase; MAPK, mitogen-activated protein kinase; MST1, mammalian sterile 20-like kinase; PINK1, PTEN-induced putative kinase 1; PKCα, protein kinase Cα; PLK, polo-like kinase; PP2A, protein phosphatase 2A; RANKL, receptor activator of NF-κB ligand; RNF, RING finger; SAPK, stress-activated protein kinase; Ser, serine; Thr, threonine; TMD, transmembrane domain; TNF-α, tumor necrosis factor-α; Ub, ubiquitin.

The BH4 domain is also necessary for BCL-2-dependent recruitment of rapidly accelerated fibrosarcoma-1 to the mitochondria, where it serves as a scaffold for BAD and protein kinase–theta interaction, leading to BAD phosphorylation, inhibiting it and consequently apoptosis [83,84]. The antiapoptotic function of BCL-2 depends on the phosphorylation of its Ser70 residue that is negatively regulated by protein phosphatase 2A-B56δ when cells are subjected to oxidative stress (Figure 5 and Table 1) [85,86].

The phosphorylation of Ser70 can be carried out by MAPK and protein kinase Cα [87,88]. In addition, p38 MAPK phosphorylates the residues threonine (Thr) 56 and Ser87 of BCL-2, decreasing its antiapoptotic activity [89]. During oxidative stress, tumor necrosis factor-α induces the dephosphorylation of these residues and leads to BCL-2 degradation [90]. Cyclin-dependent kinase (CDK) 1 was also found to phosphorylate BCL-2 at Thr56, during G_2_/M, leading to cell cycle inhibition, even in normal cells [91]. CDK1 in association with cyclin B1 also phosphorylates BCL-2 at Thr69, Ser70, Thr74, and Ser87, leading to increased affinity to BAK and BIM [92]. Furthermore, it has been reported that in BCL-2 and BCL-xL, the cleavage of the BH4 region, performed by caspase-1 or -3, can lead these proteins to promote apoptosis instead of their usual role of inhibiting it [80]. Interestingly, during exposure to Cisplatin, CDK2 was shown to phosphorylate BCL-xL at Ser73, leading to BCL-xL behaving like BAX and promoting apoptosis (Table 2) [93]. In addition, phosphorylation of the Ser62 residue or the deamidation of the asparagine residues on positions 52 and 66 in the intrinsically disordered region (IDR), also known as flexible loop domain, of BCL-xL promotes a structural rearrangement decreasing BCL-xL affinity for pro-apoptotic BH3 domains [94]. Deamidation will also promote BCL-xL degradation. It has been shown that the rate of deamidation can increase in the presence of DNA-damaging agents [95]. Two histidines close to the asparagine residues seem to be crucial for the promotion of BCL-xL deamidation by sensing pH increases caused by DNA-damaging agents [96].

The region of BCL-xL that the phosphorylated and/or deamidated IDR interacts with is also the region that interacts with p53. The interaction with p53 suppresses apoptosis dependent on BAX. Furthermore, PUMA can also bind to this region competing with p53 and BH3-only proteins, releasing them from BCL-xL [94,97]. Free p53 can then activate BAX and subsequently apoptosis [98].

BCL-xL needs to be present in the mitochondria’s outer membrane to promote its integrity. This is achieved by the interaction of BCL-xL with vacuolar protein sorting (VPS)35 and VPS26, which are components of retromer, a protein complex part of the endosomal protein sorting machinery. MICAL-like protein 1, a protein associated with retromer, is also important for BCL-xL mitochondrial localization [10]. Mitofusin 2 and mitofusin 1, two dynamin-related GTPases, also interact with BCL-xL, but only mitofusin 2 is necessary for BCL-xL-induced mitochondrial aggregation [99].

To prevent MOMP, BCL-xL inhibits BAX activity. Nonetheless, this process is still poorly understood. BAX is a pro-apoptotic BCL-2 family member that has a crucial role in the formation of pores in the mitochondrial membrane that leads to MOMP [2].

There are several proposed models for BAX and BAK, which is also involved in MOMP promotion, repression, and activation. In the direct model, BH3-only proteins can be divided in activators, such as BID and BIM, and sensitizers like BIK and PUMA [100]. For instance, when BID is cleaved by caspase-8 it converts into its active form, cBID, which contains p7 and p15 (tBID), and it translocates to the outer membrane of the mitochondria where it can activate BAX and BAK and also promote BCL-xL insertion on the membrane. However, BCL-xL inhibits BAX activation by binding to tBID or to BAX, blocking their interaction [101,102,103]. The complex formed by BCL-xL and BAX is stabilized by the interaction of vaccinia-related kinase-2A, a nuclear envelope kinase, and BCL-xL, hindering BAX dissociation while BAD binds to BCL-xL, allowing BAX and tBID interaction [104,105]. Thus, in the direct model, proteins like BAD and BIK inhibit the interaction of BCL-xL and BAX, consequently promoting apoptosis [100].

On the other hand, the indirect model proposes that BAX and BAK do not interact directly with BH3-only proteins but are indirectly activated by the inhibition of the prosurvival BCL-2 members carried by BH3-only proteins. The difference between the direct and indirect models is that in the indirect one all BH3-only proteins act as sensitizers [100]. A more recently proposed model suggests a combination of both models where prosurvival proteins can both sequester the activators and BAX and BAK [56].

BCL-xL is also involved in the retrotranslocation of BAX from mitochondria to cytosol by leading to the establishment of weak inhibitory mitochondrial complexes [106]. It is suggested that in non-apoptotic cells BAX localization in the mitochondria or cytosol follows a dynamic equilibrium that can be shifted in favor of a more cytosolic one by BCL-xL [2]. It seems that BAD plays a role in this retrotranslocation by binding to BCL-xL, promoting the release of BAX, and that AKT-mediated phosphorylation of BAD regulates this process [107]. Paradoxically, it was also found that BCL-xL overexpression led to higher concentration of BAX in the mitochondria which can possibly be explained by the fact that BCL-xL is also involved in the translocation of BAX from the cytosol to the mitochondria [2]. More recently, it was reported that binding of BCL-xL with BH3-only members inhibits its retrotranslocation, causing their accumulation in the mitochondria. It is suggested that this process might recruit pro-apoptotic proteins to the mitochondria whilst inhibiting them [108]. Additionally, it was shown that increased levels of BCL-2 and MCL-1 accelerated retrotranslocation of BAX [109]. Nonetheless, for BAK, only MCL-1 and BCL-xL increased expression led to a similar effect [110]. E2F1, a transcriptional factor, has been shown to inhibit BCL-xL retrotranslocation which seems to be essential for BCL-xL’s role in BAK inhibition [111].

Ras, a GTPase, is also involved in both pro-apoptotic and antiapoptotic signaling. In apoptosis induced by the cell membrane death receptor Fas signaling, Ras is activated. Nonetheless, active mitochondrial Ras is then regulated by BCL-2 activity, leading to the repression of Ras-mediated apoptotic signaling [112].

Both BCL-xL and BCL-2 regulate autophagy by interacting with Beclin-1, inhibiting its activity [113,114]. Beclin-1 forms, alongside other proteins, class III PI3K complexes that play a role in the formation of the autophagosome [115]. The interaction between BCL-2 and BCL-xL with BAK, BAX, and Beclin-1 can be inhibited by the JNK-mediated phosphorylation of BCL-2 and BCL-xL, leading to the promotion of apoptosis and autophagy [116]. Furthermore, in osteoclasts precursors, autophagy is induced by the receptor activator of NF-κB ligand-facilitated phosphorylation of BCL-2 Ser70 which decreases interaction between BCL-2 and Beclin-1 [82]. On the other hand, PARK2-mediated mono-ubiquitination of BCL-2 seems to enhance the interaction between BCL-2 and Beclin-1 [117].

BCL-2 is also involved in the inhibition of GABARAP, part of the GABARAP subfamily that is involved in phagophore and autophagosome formation, through the BH4 domain, affecting GABARAP lipidation and consequently inhibiting autophagy [118,119].

Other than the mitochondria, BCL-xL and BCL-2 also play a role in the ER where they are involved in the repression of inositol 1,4,5-trisphosphate receptor (IP_3_R) activity [120]. IP_3_R is a Ca^2+^ channel that is involved in important cellular events such as proliferation and apoptosis [121]. Thus, BCL-2 and BCL-xL-mediated inhibition regulates Ca^2+^ pro-apoptotic release. BCL-2 inhibits IP_3_R through the interaction of the BH4 domain with the receptor. Additionally, the transmembrane domain is essential for BCL-2 localization near the IP_3_R transmembrane domain [120,122]. However, BCL-xL-mediated inhibition of IP_3_R does not seem to be dependent on the BH4 domain but rather on BH3 domain since, when lysine (Lys) 87 was mutated, BCL-xL lost the capacity to interact with IP_3_R [120,123]. The transmembrane domain and BCL-xL capacity to dimerize are also suggested to play a role in this interaction [100]. Nonetheless, it seems that BCL-xL can both activate and inhibit IP_3_R, and the role is dependent on BCL-xL concentration. At low concentrations BCL-xL activates IP_3_R, while at higher concentrations BCL-xL inhibits it [124]. Furthermore, BCL-2 and BCL-xL inhibit ryanodine receptors in a similar way. Ryanodine receptors are Ca^2+^ channels that can be found in several types of cells, including hippocampal neurons, and it is suggested that they also play a role in apoptosis [125,126]. BCL-xL also inhibits voltage-dependent anion channel 1 (VDAC1), preventing Ca^2+^ pro-apoptotic signals from entering the mitochondria [127]. Accumulation of misfolded proteins in the ER leads to ER stress which when unresolved triggers apoptosis. It is suggested that the promotion of apoptosis during ER stress occurs through the RING finger (RNF) 183-mediated ubiquitination of BCL-xL and its consequent degradation [128].

In hippocampal neurons, BCL-xL has been shown to increase both the energy metabolism and synaptic activity while also promoting synapse formation through dynamin-related protein 1 (Drp1), a GTPase involved in neurite growth [129]. For instance, in rat hippocampal neurons, BCL-xL was shown to promote synapse formation through the regulation of caspase-3 by inhibiting Drp1-dependent mitochondrial fission [130]. In addition, BCL-xL interaction with Drp1 and clathrin is required for vesicle endocytosis regulation. This function is dependent on calmodulin which promotes the translocation of both BCL-xL and Drp1 to synaptic vesicles [131]. BCL-xL also plays a role in neurite growth by inhibiting death receptor 6 activity both under normal and hypoxic conditions [129].

BCL-2 and BCL-xL have also been shown to play a role in hematopoietic differentiation. In IL-3-deprived factor-dependent cell-Patersen Mix multipotent progenitor cells, BCL-2 led to their differentiation into granulocytes and monocytes/macrophages while the ones expressing BCL-xL differentiated into erythroid cells [132].

A role in the cell cycle has also been proposed for BCL-2 since increased levels of BCL-2 are associated with delays in the transition from G_0_/G_1_ to S phase. This delay is promoted by the regulation of reactive oxygen species (ROS) and ATP levels. It is suggested that BCL-2 can regulate mitochondrial metabolic pathways, leading to a decrease in both ATP and ROS levels while increasing p27^kip1^, a CDK inhibitor, expression [133]. It is also suggested that BCL-2 increases p130 expression and that p27 leads to CDK2 inhibition. CDK2 is involved in the degradation of both p27 and p130. p130 in turn forms a repressive complex with E2F4 that, as proposed in the model, should repress transcription of genes that are involved in cell cycle entry [134]. Moreover, BCL-2’s antiapoptotic function is inactivated through phosphorylation of Ser70 at G_2_/M through the ASK1/JNK pathway [135]. As previously referred, CDK1 also phosphorylates BCL-2 at G_2_/M. This phosphorylation occurs at Thr56, inducing G_2_/M arrest [91]. However, it has been found that BCL-2 phosphorylated at Thr56 accumulates in nuclear structures in early prophase, while in late prophase it localizes around mitotic chromosomes. In metaphase, BCL-2 remains localized around mitotic chromosomes. It seems that BCL-2 forms complexes with CDK1, nucleolin, and PP1 and that dynamic phosphorylation of BCL-2 might regulate its function during mitosis [136].

BCL-xL was also shown to play a role during the cell cycle where it undergoes dynamic phosphorylation/dephosphorylation events at both Ser49 and Ser62. A pool of Ser49-phosphorylated BCL-xL is found at centromeres during G_2_ checkpoint, a result of DNA damage. Further, another pool of phospho-BCL-xL Ser49 occurs through telophase where it seems to play a role in cytokinesis. The phosphorylation of BCL-xL is performed by polo-like kinase (PLK) 3, which is consistent with the proposed role of BCL-xL in the cell cycle since PLK3 is involved in cell cycle progression [137]. The phosphorylation at Ser62 has also been implicated in G_2_ arrest and is promoted by both PLK1 and JNK2, leading to BCL-xL recruitment to nucleolar structures during the stabilization of G_2_ arrest [138]. Additionally, PLK1 and MAPK14/p38α-mediated BCL-xL phosphorylation of Ser62 during prometaphase and metaphase led to BCL-xL’s association with the spindle assembly checkpoint silencing complexes and also to its localization at centromeres. It is suggested that BCL-xL might be involved in spindle assembly and chromosome segregation [139]. In human diploid fibroblasts this dynamic was shown to promote chromosome stability and prevent aneuploidy [140]. Furthermore, in Rat1 fibroblasts, BCL-xL was also shown to delay the cell cycle, while BAD has been shown to counter the cell cycle arrest promoted by both BCL-2 and BCL-xL [141].

BCL-xL has also been implicated in DNA damage response. JNK is activated in response to genotoxic agents, leading to its translocation to the mitochondria where it phosphorylates BCL-xL in both Thr47 and Thr115, promoting apoptosis [142]. Additionally, in rat cardiac myocyte, response to oxidative stress leads to the activation of K-Ras which consequently promotes the Ras association domain family (RASSF) 1A-mediated activation of the mammalian sterile 20-like kinase (MST) 1. MST1 in turn phosphorylates BCL-xL at Ser14, repressing its interaction with BAX, thus inducing apoptosis [143].

BCL-xL also plays a role in the regulation of mitophagy. It was found that BCL-xL interacts with PARK2, also known as Parkin, preventing its translocation to the mitochondria but also with PINK1, blocking the binding of PARK2 to PINK1, repressing mitophagy [144]. PINK1 was also found to phosphorylate BCL-xL at Ser62, promoting its antiapoptotic function [145].

**Table 1:** Post-translational modifications of BCL-2 and its functional impacts.

Modification Sites	Interacting Molecules	Modification	Functional Impacts	References
Thr56	p38 MAPK	Phosphorylation	Suppresses BCL-2 antiapoptotic activity	[89]
	CDK1	Phosphorylation	Promotes cell cycle inhibition	[91]
Thr69	CDK1 + cyclin B1	Phosphorylation	Enhanced affinity for BAK and BIM. In the presence of apigenin, suppresses BCL-2 antiapoptotic activity	[92,146]
	JNK	Phosphorylation	Probably essential for Paclitaxel to fully induce cell death. Also needed for autophagy promotion.	[116,147]
Ser70	PP2A	Dephosphorylation	Prevents BCL-2 antiapoptotic function	[86]
	MAPK	Phosphorylation	Essential for BCL-2 antiapoptotic function	[87]
	PKCα	Phosphorylation	Essential for BCL-2 antiapoptotic function	[88]
	CDK1 + cyclin B1	Phosphorylation	Enhanced affinity for BAK and BIM	[92]
	JNK	Phosphorylation	Essential for Paclitaxel to fully induce cell death. Also promotes autophagy	[116,147]
	RANKL	Phosphorylation	Promotes interaction between BCL-2 and Beclin-1	[82]
Thr74	TNF-α	Dephosphorylation	Leads to BCL-2 degradation	[90]
	CDK1 + cyclin B1	Phosphorylation	Enhanced affinity for BAK and BIM	[92]
Ser87	p38 MAPK	Phosphorylation	Suppresses BCL-2 antiapoptotic activity	[89]
	TNF-α	Dephosphorylation	Leads to BCL-2 degradation	[90]
	CDK1 + cyclin B1	Phosphorylation	Enhanced affinity for BAK and BIM. In the presence of apigenin, suppresses BCL-2 antiapoptotic activity	[92,146]
	JNK	Phosphorylation	Essential for Paclitaxel to fully induce cell death. Also promotes autophagy	[116,147]
	Paxillin	Phosphorylation	Promotes BCL-2 stability	[148]
?	PARK2	Ubiquitination	Increases BCL-2 and Beclin-1 interaction, but can also promote BCL-2 degradation	[117]

? refers to unknown modification site.

**Table 2:** Post-translational modifications of BCL-xL and its functional impacts.

Modification Sites	Interacting Molecules	Modification	Functional Impacts	References
Ser14	MST1	Phosphorylation	Represses BCL-xL and BAX interaction	[143]
Thr47	SAPK	Phosphorylation	In the presence of DNA damage promotes apoptosis	[142]
Ser49	PLK3	Phosphorylation	In G_2_ checkpoint phosphorylation of Ser49 occurs in the presence of DNA damage. In telophase Ser49 phosphorylation is implicated in cytokinesis	[137]
Asp52	?	Deamidation	Decreases BCL-xL affinity for pro-apoptotic BH3 domains. Promotes BCL-xL degradation	[94,95]
Ser62	CDK1 + cyclin B1	Phosphorylation	Suppresses BCL-xL antiapoptotic activity	[146]
	PLK1 + JNK2	Phosphorylation	Leads to BCL-xL recruitment to nucleolar structures during the stabilization of G_2_ arrest	[138]
	MAPK14 + PLK1	Phosphorylation	During prometaphase and metaphase leads to BCL-xL association with SAC silencing complexes and also to its localization at centromeres, suggesting a possible role of BCL-xL in spindle assembly and chromosome segregation	[139]
	PINK1	Phosphorylation	Prevents cell death	[145]
	PGAM5	Dephosphorylation	Prevents cell death by increasing BCL-xL affinity to BAX and BAK	[149]
Asp66	?	Deamidation	Decreases BCL-xL affinity for pro-apoptotic BH3 domains. Promotes BCL-xL degradation	[94,95]
Ser73	CDK2	Phosphorylation	Apoptosis promotion in the presence of Cisplatin	[93]
Thr115	SAPK	Phosphorylation	In the presence of DNA damage promotes apoptosis	[142]
Ser145	?	Phosphorylation	PAR2 when activated promotes phosphorylation of Ser145 stabilizing BCL-xL	[150]
?	RNF152	Ubiquitination	Leads to BCL-xL degradation	[150]
	RNF183	Ubiquitination	Leads to BCL-xL degradation	[128]
	GRIM19	Ubiquitination	Indirectly promotes BCL-xL ubiquitination leading to its degradation	[151]
	PARK2	Ubiquitination	Leads to BCL-xL degradation	[117]

? refers to unknown modification site or protein responsible for the modification.

## 4. Function of BCL-2 and BCL-xL in Cancer

### 4.1. Invasiveness and Progression

Besides their roles in non-transformed cells, BCL-2 and BCL-xL have also been reported to play roles in pro-tumoral events.

For instance, in cancer cells, BCL-2 pro-survival family members can promote cell invasion and migration by increasing ROS levels, although not high enough to stimulate cell death [152].

It has been proposed that BCL-2 regulates ROS production by modulating mitochondrial respiration in cancer cells. BCL-2 interacts with the cytochrome c oxidase (COX) Va subunit through its BH2 domain and its C-terminal region, promoting the localization of COX Va to the mitochondria. High BCL-2 expression also correlates with increased mitochondrial localization of COX Vb localization at the mitochondria, although this effect is thought to occur indirectly as a consequence of COX Va enrichment in the mitochondria. This way, BCL-2 increases COX activity and ROS production. However, under oxidative stress, BCL-2 decreases COX Vb presence in the mitochondria and stabilizes COX Va, leading to decreased COX activity and lower ROS levels [153]. In breast cancer, BCL-xL-mediated increase in ROS was shown to be dependent on BCL-xL interaction with VDAC1 [154]. Furthermore, it has been reported that BCL-xL promotion of metastasis is independent of its apoptotic activity and that the metastatic function is only observed for BCL-xL localized in the nucleus where it increased tri-methylation of histone 3 in Lys4, a marker of transcriptional activation [155]. BCL-xL translocation to the nucleus seems to be carried out by the transcriptional regulator C-terminal binding protein 2 [156].

In breast cancer, BCL-xL overexpression induces lymph node metastasis by preventing cytokine-induced cell death and promoting the ability of cells to proliferate in an anchorage-independent manner [157]. ER-positive breast cancer cells present higher expression of the lncRNA BC200. BC200 participates in breast cancer pathogenesis since it binds to *BCL-X* pre-mRNA and recruits hnRNP A2/B1 which in turn blocks SAM68 from binding to the pre-mRNA. This way, BC200 promotes the production of BCL-xL [158]. Furthermore, in triple-negative breast cancer (TNBC), high levels of the kinase Aurora A and BCL-xL were linked to promotion of metastasis [159]. In addition, BCL-2 overexpression leads to NF-κB activation, increasing matrix metalloproteinase (MMP)-9 expression in breast cancer. MMP-9 is associated with tumor metastasis and invasion [160]. It also increases MMP-2, it too associated with invasiveness, in cooperation with N-MYC, a transcription factor involved in cell proliferation, in neuroblastoma and lung cancer [161,162].

In lung cancer, BCL-xL expression is associated with migration and invasion promotion since increased expression of Let-7a-5p led to downregulation of BCL-xL and repression of these processes. Moreover, Let-7a-5p expression induces toxic autophagy by hindering the PI3K signaling pathway [163]. γ-irradiation also promoted migration and invasion in lung cancer by promoting STAT3 activity. STAT3 then induced BCL-xL transcription. Increased expression of BCL-xL was associated with augmented expression of MMP-2 and vimentin, enhanced phosphorylation of p38 and AKT, and downregulation of E-cadherin [164].

C-X-C chemokine receptor type 4 (CXCR4), a chemokine receptor, has been shown to promote tumor growth and survival by modulating microRNAs: in neuroblastoma, it downregulates miR-15a/16-1, leading to increased BCL-2 and cyclin D1 expression, while in ovarian cancer, it suppresses let-7a, resulting in the upregulation of BCL-xL [165].

Similarly, in melanoma cells, the overexpression of miR-365 led to inhibition of cell proliferation by downregulating BCL-2 and cyclin D1 [166]. BCL-2 is also involved in progression in melanoma by promoting stability of Semaphorin 5A, an axon regulator, at the mRNA and protein levels. Semaphorin 5A promotes migration through the activation of the MEK/ERK pathway [167]. The lncRNA LHFPL3-AS1-long also plays a role in melanoma stem cells tumorigenesis by binding to miR-181a-5p, preventing BCL-2 mRNA degradation [168]. Moreover, metastatic melanoma patient samples showed higher expression of BCL-2 and BCL-xL than primary melanoma, benign nevi, and normal skin samples, suggesting a role for these proteins in melanoma progression [169]. Accordingly, BCL-2 overexpression was found to be associated with higher expression of MMP-2 and MMP-7 [170]. In melanoma and glioblastoma cells, BCL-xL overexpression was found to promote migration, invasion, and angiogenesis [171].

Furthermore, glioblastoma progression is promoted in part by increased expression of BCL-xL caused by downregulation of tumor suppressor candidate 2 due to neural precursor cell-expressed developmentally downregulated 4-mediated polyubiquitination [172]. Accordingly, inhibition of the splicing factor SF3B1 reduced migration, tumorigenesis, and vascular endothelial growth factor (VEGF) secretion by shifting *BCL-X* pre-mRNA for BCL-xS production and repressing the AKT/mTOR/β-catenin pathways [71]. Phosphatase and tensin homolog (PTEN), in normal cells, presents tumor suppressive activity. However, in glioblastoma cells with mutant p53, PTEN was shown to interact with the complex formed by mutant p53, acetyl-CBP, and NFYA, promoting their binding to BCL-xL and c-Myc promoter regions upregulating these proteins. Consequently, the upregulation of BCL-xL and c-Myc promoted cell invasion, proliferation, and tumor progression [173]. Moreover, BCL-2 expression in glioma cells increases Furin and transforming growth factor-β (TGF-β) expression which in turn leads to the upregulation of MMPs, promoting glioma cells invasiveness [174]. Similarly, BCL-xL upregulation induced TGF-β2, MMP-2, and MT1-MMP, promoting invasiveness of malignant glioma cells [175].

BCL-2 overexpression also promotes migration through modulation of urokinase-type plasminogen activator receptor (uPAR). uPAR is the receptor of uPA, a protease that converts the extracellular zymogen plasminogen to plasmin. Plasmin is involved in invasion and metastasis. By activating ERK leading to increased Sp1, a transcriptional factor, activity, BCL-2 promotes uPAR expression and consequently invasion [176]. BCL-2 was also shown to promote cell migration through interference in the Hippo pathway since it reduces MST2 protein levels. MST2 regulates Yes-associated protein (YAP), a transcription coregulator, by activating large tumor suppressor kinases (LATS) 1/2. LATS1/2 activation leads to the maintenance of YAP in the cytoplasm, inhibiting its transcriptional activity [177]. Microphthalmia-associated transcription factor (MITF) is a transcription factor involved in the expression of miR-211, a microRNA, associated with migration and invasion suppression, that is regulated by BCL-2. BCL-2 represses MITF nuclear activity probably through the interaction with heat shock protein (HSP) 90 [178]. In addition, both BCL-2 and BCL-xL can bind to a tumor suppressor protein known as SUFU, repressing its binding to GLI proteins. GLI proteins regulate some cell proliferation genes’ expression, and their binding to SUFU suppresses this activity, decreasing cell proliferation [179].

Circular RNAs have also been reported to contribute to cancer progression since, in osteosarcoma, circ_0000376 was found to bind to miR-432-5p, allowing the expression of BCL-2. Inhibition of circ_0000376 led to the repression of osteosarcoma cells migratory and invasive capabilities and increased cell death [45].

The knockdown of BCL-2, BCL-xL, or MCL-1 hindered migration and invasion in colorectal cancer (CRC) cells [180]. Moreover, BCL-2 was found to be associated with colon carcinogenesis since overexpression of miR-15a, which regulates BCL-2 expression, led to lower cell proliferation and reduced cell invasion properties [181]. Additionally, BCL-2 expression has been associated with early stages of carcinogenesis in colorectal neoplasias [182]. It has also been reported that BCL-2 was upregulated in CRC metastatic cells when compared with nonmetastatic ones [183]. Furthermore, dual specificity phosphatase 4 (DUSP4) is also associated with carcinogenesis in CRC. DUSP4 prevents JNK-mediated phosphorylation of BCL-2, while its silencing blocks the interaction of BCL-2 with Beclin-1 or BAX, increasing autophagy and apoptosis and repressing migration and invasiveness [184]. Depletion of circDUSP16 also affected CRC cells’ migration, invasion, and proliferation due to decreased BCL-2 expression. circDUSP16 targets miR-432-5p, and its depletion allows miR-432-5p to downregulate E2F6. E2F6 overexpression was previously reported to be associated with c-Src/ERK and BCL-2 upregulation. Thus it seems that E2F6 promotes BCL-2 expression by inducing c-Src/ERK signaling [185,186]. Besides BCL-2 overexpression in CRC, BCL-2 Ser70 phosphorylation status was also related with tumor aggressiveness since tumors in more advanced stages showed lower expression of phosphorylated Ser70 [187]. BCL-xL is also involved in CRC cells’ invasion, proliferation, and clonogenic formation since its inhibition impaired all of these processes [188]. The protease-activated receptor 2 (PAR2) is also involved in cancer progression and in the regulation of the immune microenvironment. PAR2 stabilizes BCL-xL by promoting Ser145 phosphorylation. The phosphorylation of this residue prevents interaction with RNF152 and consequently BCL-xL polyubiquitination and degradation. Expression of BCL-xL inhibits type I IFN secretion, preventing recruitment of CD8^+^ T cells to metastatic sites [150,189]. Additionally, the lncRNA HEIH is involved in CRC tumorigenesis by targeting miR-939, preventing repression of BCL-xL transcription since miR-939 binds to NF-κB, blocking its binding to *BCL-X* promoter [190].

In oral cancer several studies have shown that BCL-2 is associated with differentiation since BCL-2 expression is increased in sequentially progressing epithelial dysplasia and in poorly differentiated carcinomas when compared to well-differentiated ones [191,192,193,194,195]. In accordance, Niedzielska et al. showed that in patients with squamous cell carcinoma (SCC), BCL-2 is more expressed than in patients with hyperplasia and patients with neoplasm in situ malignancy [196]. Nevertheless, tissue from the incision line, close to the tumor, showed higher BCL-2 expression in the three groups assessed. Similar results were reported by Juneja et al. [197]. However, in some studies this relation was not observed, with BCL-2 being sporadic in oral premalignant tissue [198,199]. On the other hand, it has been reported that there is no significant difference regarding BCL-xL expression between poorly differentiated oral squamous cell carcinoma (OSCC) and basaloid SCC [200]. Nonetheless, BCL-xL expression in OSCC is correlated with progression and resistance to Cisplatin treatment [201]. Overexpression of BCL-2 in OSCC was also found to lead to increased expression of MMP-9 and enhanced migration and invasion behavior [202]. Moreover, overexpression of BCL-2 and p53 seems to be mutually exclusive, suggesting that both genes can induce carcinogenesis in OSCC, independently [203]. Nonetheless, tumors expressing both proteins showed higher probability of presenting unfavorable characteristics [204]. Another study found higher mRNA ratios of BCL-2/BAX mostly in poorly differentiated oral carcinomas, while BAX at the protein level was downregulated in these poorly differentiated carcinomas [205]. The downregulation of BCL-2 by miR-34a was also found to inhibit sinonasal SCC migration and invasion capabilities [43].

The BCL-2 antiapoptotic family members have been found to contribute to leukemogenesis promoted by human T-cell leukemia virus type 1 and bovine leukemia virus. These viruses upregulate BCL-2, BCL-xL, MCL-1, and BFL-1 while they downregulate BAX, BIM, and BID. This way infected lymphocytes can survive and proliferate, increasing genomic instability and promoting leukemogenesis [206].

In acute myeloid leukemia (AML), spastic paraplegia 6 protein, a dominant autosomal HSP, is associated with disease progression since it regulates the BMPR2-SMAD-BCL-2/BCL-xL pathway [207]. Furthermore, coexpression of MYC and BCL-xL or BCL-2 can drive AML tumorigenesis [208].

In cervical cancer, tumorigenesis was found to be promoted by the lncRNA RUSC1-AS1 since it acted as a CeRNA, inhibiting miRNA-744 and leading to increased expression of BCL-2 [41].

In retinoblastoma, a correlation between BCL-2 expression and tumor invasiveness and poor differentiation was found [209].

In hepatocellular carcinoma (HCC), follistatin-like protein 5 was shown to inhibit cancer progression by downregulating BCL-2 and upregulating BAX, BAD, and PUMA [210]. Similarly, both miR-202 and miR-448 were also found to repress HCC progression and cell growth by targeting BCL-2 [42,44].

BCL-xL is also involved in prostate cancer progression since a study showed that the overexpression of miR-608 which targets BCL-xL mRNA represses cancer progression, while another study demonstrated that overexpression of BCL-xL increases cancer progression by repressing senescence and apoptosis, reducing survival in vivo [211,212].

BCL-xL expression was also found to be necessary to promote islet tumor cells and pancreatic neuroendocrine cancer invasiveness [213,214].

Progression in cholangiocarcinoma is associated with aberrant alternative splicing. For instance, high expression of serine/arginine protein kinase (SRPK) 1 and SRPK2 is usually found on this type of cancer, leading to phosphorylation of SRSFs that promote antiapoptotic splicing isoforms such as MCL-1 and BCL-xL. Consequently, inhibition of SRPK1 and SRPK2 led to increased expression of MCL-1S and BCL-xS, promoting cell death [215].

PARK2 is a tumor suppressor that regulates the cell cycle and programmed cell death. In several cancers, PARK2 is usually dysregulated, resulting in increased proliferation and repression of apoptosis. It was found that PARK2 can ubiquitinate BCL-xL, and this regulation is essential for PARK2 tumor suppression activity. Since PARK2 promotes BCL-xL degradation, the dysregulation of PARK2 leads to high expression of BCL-xL and to cancer cell death prevention [117].

Both BCL-2 and BCL-xL play critical roles in tumor proliferation and invasiveness which are related with more aggressive tumors. Thus, BCL-2 and BCL-xL can be important targets in the treatment of more advanced tumors.

### 4.2. Angiogenesis

Angiogenesis is an important process for cancer cells since it is essential for cell growth and metastasis, and BCL-2 and BCL-xL have been found to regulate this process in several types of cancer. For instance, overexpression of BCL-xL leads to increased levels of IL-8, through NF-κB activation, and consequently to the promotion of angiogenesis in melanoma and glioblastoma cells [216,217]. Similarly, in a zebrafish melanoma xenograft model, BCL-xL was also found to promote angiogenesis through the regulation of IL-8 [218]. Additionally, BCL-2 overexpression enhances VEGF expression by promoting PI3K and MAPK pathways signaling in cancer cells subjected to hypoxic conditions [219,220]. Similarly, in melanoma and breast cancer cells, increased BCL-2 expression also led to induction of angiogenesis by modulation of VEGF expression through HIF-1 and by stabilization of VEGF mRNA [221,222]. In lymphoma, overexpression of BCL-2 isoform β was shown to promote angiogenesis by interacting with the chaperonin T-complex protein ring complex essential for the secretion of VEGF-A and vessel tube formation [223]. In colon cancer, BCL-2 expression was reported to be correlated with VEGF expression and microvessel density which potentially suggests a role for BCL-2 in angiogenesis in this type of cancer [224].

In HCC, BCL-2 was found to interact with the transcription factor Twist1 through both its BH2 and transmembrane domains. Under hypoxic conditions these proteins are coexpressed. Their interaction increases Twist1 nuclear presence, inducing the expression of several genes such as VEGFR1, VEGFR2, MMP-2, and MMP-9 involved in processes like angiogenesis and epithelial–mesenchymal transition [225].

Angiogenesis is essential to provide oxygen and nutrients to cancer cells needed for their growth but also allows cells to escape to the bloodstream and invade other tissues. Thus, it is not surprising that BCL-2 and BCL-xL play a role in the regulation of angiogenesis since they are so important for cell invasiveness.

### 4.3. BCL-2 and BCL-xL Effects on Drug Responses

BCL-2 and BCL-xL are not only involved in processes vital for cancer cell survival and tumor progression but also in the resistance to treatment. For instance, BCL-2 and BCL-xL overexpression has been shown to lead to radioresistance and chemoresistance to several drugs, including Cytosine arabinoside, Cisplatin, Topotecan, Gemcitabine, Docetaxel, and Paclitaxel [226,227,228,229,230,231,232,233,234,235,236,237,238,239,240,241,242,243].

In advanced bladder cancer patients previously treated with radiotherapy, lower expression of BCL-2 led to better survival after treatment with Cisplatin than in patients with high BCL-2 expression [244]. Additionally, BCL-2 expression was associated with worse response to treatment with concurrent radiotherapy and platinum therapy in advanced oropharyngeal SCC. BCL-2, but not BCL-xL, expression was also found to induce resistance in vitro [245].

In gallbladder cancer and osteosarcoma cell lines, miR-125b was found to increase sensitivity to Cisplatin by downregulating BCL-2 [246,247]. Similarly, miR-204 also conferred sensibility to Cisplatin in neuroblastoma [248]. On the other hand, nicotine was found to increase resistance to Cisplatin by increasing BCL-2 expression in oral cancer [249]. In ovarian cancer, inter-α-trypsin inhibitor heavy chain 3, involved in the stabilization of the extracellular matrix, downregulation increases BCL-2, BCL-xL, and MCL-1 expression after Cisplatin treatment, leading to resistance to this drug [250]. Similarly, in bladder cancer, the downregulation of genes associated with retinoid-interferon-induced mortality-19 also induced Cisplatin resistance by reducing BCL-xL polyubiquitination and degradation [151]. Suppression of BCL-xL deamidation has also been found to lead to resistance to DNA-damaging agents such as Cisplatin [251,252]. Cisplatin treatment in gastric cancer cells increased expression of CDK1 which in turn activated DNA methyltransferase 1, silencing miR-145. miR-145 suppression increases SRY-box transcription factor 9 (SOX9) expression and consequently BCL-xL expression since BCL-xL is a direct transcriptional target of SOX9. Increased expression of BCL-xL decreases gastric cancer cells sensitivity to Cisplatin [253]. Additionally, overexpression of miR-193a-3p in CD44^+^ gastric cancer cells leads to increased expression of BCL-xL and resistance to Cisplatin by targeting SRSF2 [254].

In mesothelioma cell lines, inhibition of both BCL-xL and BCL-2 increased cell sensitivity to Cisplatin [255]. However, expression of BCL-xL was found to only confer resistance to Cisplatin in head and neck squamous cell carcinoma (HNSCC) cells with wild type p53 since HNSCC cells with mutant p53 were sensitive to Cisplatin regardless of BCL-xL expression [235].

Moreover, knockdown of BCL-2, BCL-xL, or MCL-1 enhanced CRC cells’ sensitivity to Oxaliplatin [180]. Nonetheless, Oxaliplatin apoptosis induction occurs due to a shift in splicing from BCL-xL to BCL-xS. Oxaliplatin weakens 14-3-3ε binding to SRSF10 which dissociates from hnRNP F/H, but not hnRNP K, resulting in its dissociation from the pre-mRNA. 14-3-3ε continuous interaction with hnRNP A1 and Oxaliplatin-induced dephosphorylation of SAM68 enhance the affinity of SAM68 to hnRNPA1 which then represses RNPS1, thus promoting BCL-xS production [256]. Gfi1, a transcriptional repressor, was also shown to prevent DNA damage-induced apoptosis by repressing the transcription factor PU.1, averting Hemgn degradation. Hemgn then activates the *BCL-X* promoter, upregulating BCL-xL [257].

Breast cancer cell lines overexpressing BCL-2 showed increased resistance to Cisplatin and Bischloroethylnitrosourea while showing higher sensitivity to Doxorubicin, Vincristine, Vinblastine, and Actinomycin D [258].

On the other hand, in a non-Hodgkin lymphoma cell line, increased expression of HIF-1α led to resistance to treatment with Cisplatin and Doxorubicin by promoting BCL-xL expression [259]. Likewise, high expression of BCL-xL in HCC cells induces resistance to Doxorubicin. Interestingly, Pyrrolidine dithiocarbamate, an antioxidant that can also inhibit NF-κB, overcame this resistance by inducing paraptosis [260]. A chronic myeloid leukemia (CML) CD44v16 cell line was also found to be resistant to Doxorubicin through the activation of the NF-κB/Snail/BCL-2 pathway [261].

Furthermore, BCL-2 positive non-small cell lung cancer (NSCLC) patients were found to be less responsive to treatment with Cisplatin combined with Gemcitabine than BCL-2 negative ones [232]. Similarly, in resistant non-Hodgkin lymphoma, low expression of BCL-2 was predictive of a better response to treatment with Gemcitabine in combination with Cisplatin and Dexamethasone [262].

BCL-xL has been associated with Gemcitabine resistance and the addition of DT2216, a BCL-xL degrader, overcame Gemcitabine resistance both in vitro and in PDTX pancreatic cancer models [233]. Moreover, pancreatic carcinoma cells with higher BCL-2 expression showed a higher Gemcitabine 50% lethal dose than cell lines with lower BCL-2 expression [263].

One of the most common TNBC treatment options is the combination of Doxorubicin, Cisplatin, and 5-fluorouracil (5-FU). Nonetheless, in a TNBC cell line, resistance to this combinatorial approach arose from the expression of BCL-xL [264].

In CRC, paxillin, an adapter protein, was found to phosphorylate BCL-2 at Ser87, promoting its stability and leading to resistance to 5-FU [148]. BCL-xL expression was also found to confer resistance to 5-FU and radiotherapy in CRC, while inhibition of BCL-xL combined with 5-FU or radiotherapy led to synergistic effects [265]. In gastric carcinoma, miR-383 was shown to increase cell sensitivity to 5-FU by targeting BCL-2 mRNA [50]. Additionally, high expression of phosphatase phosphoglycerate mutase family member 5 (PGAM5) in HCC patients leads to resistance to treatment with 5-FU due to PGAM5 and BCL-xL interaction that promotes BCL-xL stabilization [266].

The mechanism behind BCL-2-induced resistance to DNA-damaging drugs such as Cisplatin and 5-FU is related to the Ser70 phosphorylation of BCL-2. The phosphorylation of this residue decreases the affinity of BCL-2 to the mitochondrial complex-IV subunit-5A. This interaction is essential for mitochondrial complex-IV activity and ROS production. Since Etoposide and Doxorubicin led to the reduction in Ser70 phosphorylation, resistance to these drugs was not observed [267].

Furthermore, glycochenodeoxycholate, the principal compound in the bile, also promotes BCL-2 Ser70 phosphorylation, increasing HCC cell survival and promoting chemoresistance [87]. Active GTPase-Rac1 was also found to promote BCL-2 phosphorylation at Ser70, preventing apoptosis of cancer cells [268].

On the other hand, in breast cancer, PARK2 expression confers sensitivity to antimicrotubule agents such as Docetaxel and Vinorelbine by interacting with BCL-2 phosphorylated in Ser70, promoting BCL-2 polyubiquitination and consequently its degradation. Treatment with these drugs leads to PARK2 upregulation which can then lead to degradation of BCL-2, inducing BAX activation and apoptosis [269]. Nonetheless, Docetaxel resistance in prostate cancer was shown to arise from TGF-β induction of Krüppel-like factor 5, a transcription factor, acetylation which in turn leads to BCL-2 upregulation. Moreover, TGF-β also prevents BCL-2 ubiquitination induced by Docetaxel [270].

It was also reported that phosphorylation of Thr69, Ser70, and Ser87 of BCL-2 occurs after Paclitaxel treatment. However, contrarily to the effects observed for other drugs, it is suggested that at least phosphorylation of both Ser70 and Ser87 is essential for Paclitaxel to fully induce cell death [147]. Furthermore, in breast and ovarian cancer cells, overexpression of miR-203b-3p and miR-203a-3p increased sensitivity to Paclitaxel by downregulating BCL-xL. Interestingly, c-Myc was found to promote the transcription of miR-203b-3p and miR-203a-3p in breast cancer cells [75]. Moreover, antimicrotubule agents cause cell death by inducing prolonged mitotic arrest. Cell fate of mitotic arrested cells is defined by the duration of the arrest, cyclin B1 degradation, and apoptotic signaling. For instance, high expression of BCL-xL allows for cyclin B1 degradation to reach it threshold, and the cell exits mitosis, while low expression of BCL-xL will lead to the apoptotic signaling threshold to be reached and cell death induced. An intermediate level of BCL-xL might let the cell exit mitosis but die after. Thus, BCL-xL expression can influence the sensitivity to drugs that act by causing mitotic arrest [271,272].

In cancer cells treated with Vinblastine, PGAM5 dephosphorylates BCL-xL at Ser62, increasing BCL-xL’s affinity to BAX and BAK to prevent cell death [149].

In CRC, PAR2/BCL-xL axis is involved in epidermal growth factor receptor (EGFR) targeting resistance [150]. In addition, in *EGFR*-mutant lung cancer, deficiency of RBM10 was found to reduce sensitivity to EGFR targeting by reducing the BCL-xS/BCL-xL ratio [273]. Similarly, Gefitinib-resistant lung cancer cells were shown to inhibit autophagy through SRSF1 activity, which promotes production of the BCL-xL isoform. BCL-xL binds to Beclin-1 and prevents autophagy. On the other hand, under starvation conditions, SRSF1 is repressed increasing the BCL-xS/BCL-xL ratio. Beclin-1 is then free to interact with PIK3C3 and induce autophagy [274].

Furthermore, abnormal splicing of *BCL-X* was found to confer resistance to Imatinib in CML cells by decreasing BCL-xS/BCL-xL ratio. Restoring *BCL-X* splicing sensitized CML cells to Imatinib both in vitro and in vivo [275]. CML cells resistant to Imatinib were also found to overexpress methyltransferase-like 14 (METTL14). METTL14 increases the m6A level at the A1001 site of the *BCL-X* mRNA which is then recognized by hnRNP C to induce BCL-xL expression, promoting CML progression and Imatinib resistance. Additionally, METTL4 overexpression also leads to upregulation of BCL-2 and downregulation of BAX and caspase-3 [276].

Cancer cells’ sensitivity to the splicing modulator E7107, which targets SF3b, decreased in the presence of BCL-xL. Nonetheless, no effect was observed for splicing modulator targeting SRPK or RBM39/DCAF15 potentially due to the fact that these proteins do not play a role in the splicing regulation of *BCL-X* mRNA [277].

In a multiple myeloma cell line with antisense p53, increased expression of BCL-2 was correlated with Dexamethasone resistance [278]. Furthermore, the expression of BCL-2 in diffuse large B-cell lymphoma (DLBCL) patients treated with Cyclophosphamide, Doxorubicin, Vincristine, and Prednisone is associated with worse prognosis. However, addition of Rituximab to this treatment approach overcame the association of BCL-2 expression to worse prognosis [279]. In multiple myeloma, BCL-xL expression seems to be associated with resistance to treatment with Melphalan and Prednisone or Vincristine, Adriamycin, and Dexamethasone [280].

In nonmuscle invasive bladder cancer, it was shown that protein S100A16 promotes resistance to Mitomycin C by increasing AKT/BCL-2 pathway signaling [281].

In AML, induction of resistance to Cytarabine is suggested to be promoted by CXCR4-facilitated repression of Let-7a expression, which induces Yin Yang 1-mediated transcriptional promotion of MYC and BCL-xL [282]. Paradoxically, a different study showed that activation of CXCR4 led to downregulation of BCL-xL and upregulation of NOXA and BAK [283].

GEX1A is a splicing modulator that in leukemic cells led to cell death by shifting MCL-1 splicing towards the pro-apoptotic isoform MCL-1S. Nonetheless, cells with high levels of BCL-xL were less responsive to GEX1A treatment [284]. Moreover, in HCC cell lines, increased expression of Let-7c, a miRNA targeting BCL-xL, potentiated the apoptotic effect of Sorafenib, a MCL-1 inhibitor [76].

In AML, administration of Flavopiridol, a CDK inhibitor, increases BCL-2 expression, and it is suggested that BCL-2 inhibition could enhance Flavopiridol efficacy [285].

High expression of BCL-xL in glioma stem cells has also been associated with increased resistance to treatment with Volasertib, a PLK1 inhibitor [286].

In TNBC, inhibition of BCL-xL synergized with CDK1/2/4 inhibitors, but not with inhibitors of the transcription factor Forkhead box M1, CDK4/6, Aurora A, and Aurora B [287]. Nonetheless, in small cell lung cancer (SCLC), resistance to the Aurora B inhibitor AZD2811 was overcome by the inhibition of BCL-2 [288]. Moreover, BCL-2 and BCL-xL were found to prevent death of MYC overexpressing cells prompted by the pan-Aurora inhibitor VX-680, but not polyploidy induction. This is achieved by the interaction of BCL-2 and BCL-xL with Beclin-1 and autophagy-related gene 6 blocking autophagy induction [289].

High expression of BCL-xL has also been associated with resistance to v-Raf murine sarcoma viral oncogene homolog B (BRAF) inhibitors. BRAF is involved in the regulation of cell growth, survival, and differentiation, and its inhibition in metastatic melanoma leads to increased expression of several BCL-2 family proteins such as BCL-xL and BCL-w. Furthermore, high BCL-2 expression before treatment was inversely correlated with response to BRAF inhibition [290].

In mantle cell lymphoma, depletion of BAX and overexpression of BCL-xL both alone and in conjugation render cells resistant to treatment with the proteasome inhibitor Bortezomib [291].

Argininosuccinate synthetase 1 silencing is common in several types of cancer, resulting in dependency on extracellular arginine. In this sense, arginine deprivation therapies have been explored but have shown disappointing anticancer effects. This can be explained by the fact that BCL-xL prevents apoptosis induced by this type of therapies [292].

In myeloma, high expression of BCL-2 is associated with interferon therapy resistance [293]. Moreover, lower expression of BCL-2 predicts worse prognosis in adjuvant endocrine therapy-treated ER-positive breast cancer patients [294].

ER stress inducers, in HCC, led to the upregulation of GOLGA2P10, a lncRNA, and consequently to increased BCL-xL expression and BAX phosphorylation preventing cell death [295].

Furthermore, in PDAC, collagen XI/αI is associated with resistance to treatment by inducing the AKT/CREB/BCL-2 pathway, which leads to increased expression of BCL-2 and decreased activity of BAX, thus inhibiting apoptosis [296].

The RNA helicase DHX33 in association with activating protein 2β was shown to promote cancer cell survival by increasing BCL-2 mRNA expression [297].

In CRC, RASSF4 expression is usually downregulated. RASSF4 regulates BCL-2 expression through YAP and its lower expression in CRC induces cell proliferation and drug resistance [298].

CML cells expressing BCR-ABL were shown to repress apoptosis induced by chemotherapy through the promotion of STAT5 activity that leads to increased expression of BCL-xL [299,300].

Moreover, in melanoma, expression of BCL-2 was associated with resistance to biochemotherapy by repressing apoptosis induction [301].

In patients with operable carcinoma of the breast, low BCL-2 expression is predictive of pathological complete response after preoperative chemotherapy [302].

However, BCL-2 expression can also confer sensitivity to some drugs. For instance, high expression of BCL-2 in non-germinal-center B-cell-like DLBCL was also associated with better response to Zanubrutinib, a Bruton tyrosine kinase inhibitor [303]. Moreover, postmastectomy radiotherapy in breast cancer patients led to a better outcome in high BCL-2 expression patients [304]. Similarly, in HNSCC, BCL-2-positive patients also had better response to radiotherapy than BCL-2 negative patients [305].

However, BCL-2 positive prostate cancer patients showed increased failure to radiotherapy treatment. Additionally, the group who showed worse failure to treatment was the BCL-2 positive group with abnormal expression of BAX [306]. In human glioma, miR-153-3p was found to increase radiosensitivity by targeting BCL-2 expression [307]. Treatment with the BCL-xL inhibitor A1331852 also radiosensitized mesothelioma cells [308]. Accordingly, in laryngeal cancer, expression of both BCL-2 and BCL-xL was associated with radioresistance [309]. In CRC overexpression of tumor necrosis factor receptor-associated factor 4, a E3 ligase, led to radioresistance through the activation of JNK/c-Jun and consequent increase in BCL-xL expression [310]. Moreover, in malignant glioma and pancreatic cells, BCL-xL expression was also associated with radioresistance [311,312]. In prostate cancer patients, BCL-2 was found to be overexpressed in patients who failed brachytherapy compared to patients who responded to treatment [313].

Prostate cancer patients BCL-2 negative and with normal BAX showed high response to treatment with androgen deprivation combined with radiotherapy. The predictive value of negative BCL-2 and normal BAX was more pronounced for short-term androgen deprivation than for long-term [314]. On the other hand, BCL-2-positive prostate cancer patients treated with neoadjuvant androgen deprivation and radical radiotherapy showed better prognosis than BCL-2-negative ones [315].

Furthermore, prostate cancer cells overexpressing BCL-2 are susceptible to Poly (ADP-Ribose) polymerase (PARP) inhibition combined with radiotherapy. Overexpression of BCL-2 blocks Ku80 from entering the nucleus. Ku80 is essential for the non-homologous end joining DNA repair pathway. Thus, BCL-2 overexpressing cells depend on the alternative PARP1-dependent end-joining pathway to repair DNA double-strand breaks, making them susceptible to PARP inhibition [316].

Another common mechanism that allows cancer cells to survive therapy is through the induction of senescence. For instance, in melanoma cells, a combination of an inhibitor of Aurora A, which promotes senescence, and a BCL-xL inhibitor led to enhanced treatment efficacy. BCL-xL represses BAX, maintaining p53 activation which in turn promotes p21-mediated senescence. When BCL-xL is inhibited p21 is degraded by caspases and the apoptotic pathway is induced [317]. Accordingly, BCL-xL has been described to promote senescence in several types of cancer [56].

Treatment with Palbociclib or bromodomain and extra-terminal (BET) protein inhibitors can induce senescence, leading to resistance to these drugs. For instance, Palbociclib-induced melanoma senescent cells showed a reduction in HRK and BIM expression and increased BCL-xL:BAK affinity, preventing apoptosis [318]. Similarly, TNBC BET inhibitors-induced senescent cells present higher levels of BCL-xL, and its inhibition leads to increased sensitivity to BET inhibitors [319].

Moreover, in PDAC, BCL-xL was found to protect cells exposed to a microenvironment scarce in oxygen and nutrients by repressing cell cycle progression [320]. An acidic microenvironment also leads to the upregulation of BCL-2 and BCL-xL by MEK/ERK activity promoted by G protein-coupled receptor 65 which acts as an acid sensor [321]. Overexpression of BCL-2 and BCL-xL is largely linked to increased resistance to chemotherapy and radiotherapy. Therefore, combining standard treatments with BCL-2 and BCL-xL inhibitors may help overcome this resistance and improve drug efficacy and patient outcomes.

## 5. BCL-2 and BCL-xL Expression in Cancer Across TCGA and CPTAC Datasets Using UALCAN Analysis

Given the central roles of BCL-2 and BCL-xL in cancer-related processes and drug resistance, we used the UALCAN webtool to assess BCL-2 and BCL-xL mRNA and protein expression levels across multiple cancer types compared with normal tissues (Table 3 and Table 4). At the mRNA level, BCL-2 upregulation was observed in all kidney cancer types analyzed, as well as in cholangiocarcinoma and HCC, but no upregulation was observed at protein level. Conversely, BCL-2 mRNA was downregulated in bladder urothelial carcinoma, breast invasive carcinoma, cervical SCC, colon adenocarcinoma, uterine corpus endometrial carcinoma, lung SCC, prostate adenocarcinoma, rectum adenocarcinoma, stomach adenocarcinoma, and thyroid carcinoma, while at the protein level it was downregulated in breast cancer and HCC.

On the other hand, BCL-xL was upregulated in bladder urothelial carcinoma, breast invasive carcinoma, cervical SCC, cholangiocarcinoma, colon adenocarcinoma, uterine corpus endometrial carcinoma, esophageal carcinoma, HNSCC, kidney chromophobe, renal papillary cell carcinoma, HCC, prostate adenocarcinoma, rectum adenocarcinoma, stomach adenocarcinoma, and thyroid carcinoma at the mRNA level and in ovarian and colon cancers, uterine corpus endometrial adenocarcinoma, lung adenocarcinoma, and pancreatic adenocarcinoma at the protein level. Conversely, BCL-xL was downregulated at the mRNA level in lung SCC, and at the protein level in clear cell renal cell carcinoma, lung SCC, and HNSCC.

Nonetheless, it is important to note that the UALCAN webtool does not distinguish between BCL-xL and BCL-xS isoforms, as the search is based on the gene (*BCL2L1*) and not the protein.

## 6. Co-Targeting of BCL-2 and BCL-xL in Clinical Trials

Due to their roles in cancer progression and also in resistance to chemo- and radiotherapy, several dual BCL-2/BCL-xL inhibitors have been designed and assessed in clinical trials across a wide spectrum of malignancies, including HCC, lung cancer, melanoma, and ovarian cancer (Table 5) [322].

Navitoclax (ABT-263), a BH3-mimetic that targets BCL-2, BCL-xL, and BCL-w, has been the most extensively investigated agent of this class. Early phase I trials in hematologic and solid tumors demonstrated antitumor activity with manageable toxicity profiles [323,324,325].

In SCLC and other solid tumors, Navitoclax induced partial responses or stable disease in a minority of patients, though severe adverse events (AEs) such as fatal respiratory failure, left ventricular systolic dysfunction, and asymptomatic lipase elevation were occasionally reported [308]. In relapsed or refractory CLL, partial responses were observed, but hematologic toxicities, especially thrombocytopenia and neutropenia, were frequent, establishing a maximum tolerated dose (MTD) of 200 mg/day with intermittent dosing [309].

A phase 2 study in patients with relapsed/refractory lymphoid malignancies treated with Navitoclax corroborated these findings even though clinical activity was only observed in a minority of these patients [326]. Nonetheless, two phase 2 trials showed that Navitoclax alone had limited activity against advanced and recurrent SCLC and ovarian cancer [327,328].

Combinatorial approaches with Navitoclax have also been investigated, and in patients with solid tumors the combination of Gemcitabine and Navitoclax was deemed safe but produced mostly stable disease, while the combination of Navitoclax and Docetaxel was also tolerable and showed clinical activity with partial responses in a subset of patients [329,330].

Navitoclax plus Trametinib, a MEK inhibitor, was also deemed safe and led to durable responses, and a recommended phase II dose was established [331].

The phase II recommended dose could not be achieved with the combination of Navitoclax and Erlotinib while a study investigating Navitoclax with Cisplatin and Paclitaxel was discontinued due to high toxicity and low clinical activity [332,333]. Nonetheless, Navitoclax with Paclitaxel showed moderate activity. The addition of irinotecan to Navitoclax resulted in partial responses. However, grade ≥ 3 AEs occurred in 77.4% of patients [334].

In NSCLC, Navitoclax combined with Osimertinib, an EGFR inhibitor, was also tolerable with clinical efficacy [335]. Early studies pairing Navitoclax with Vistusertib, an mTOR inhibitor, or Sorafenib, a multi-kinase inhibitor, also indicated manageable safety but minimal objective responses [336,337].

In a phase 2 study with patients with myelofibrosis, Navitoclax combined to Ruxolitinib, an inhibitor of Janus kinase 1 and 2, led to significantly improved spleen volume and symptom burden even though the median overall survival (OS) was not met [338]. A phase I trial in relapsed/refractory CD20+ lymphoid malignancies exploring the combination of Navitoclax with Rituximab, a CD20 inhibitor, demonstrated multiple complete and partial responses, even though grade 4 thrombocytopenia occurred in 17% of patients [339]. In B-cell CLL, the same combination was well tolerated and led to higher response rates and prolonged progression-free survival [340]. Navitoclax with Venetoclax and chemotherapy in relapsed/refractory acute lymphoblastic leukemia and lymphoblastic lymphoma achieved objective responses but were limited by grade 3/4 myelosuppression [341].

Additional trials in different cancer types evaluating Navitoclax with other agents, including Ketoconazole (NCT01021358), Etoposide plus Cisplatin (NCT00878449), Olaparib (NCT05358639), Rifampin (NCT01121133), Venetoclax plus cladribine-based salvage therapy (NCT06007911), Venetoclax with Decitabine (NCT05455294, NCT05222984, NCT05740449), Venetoclax (NCT05215405, NCT05192889, NCT05054465), Ruxolitinib with or without Mivebresib (NCT04041050, NCT04480086), Ruxolitinib plus ABBV-744 (NCT04454658), Ruxolitinib (NCT04472598, NCT04468984), Fludarabine plus Cyclophosphamide and Rituximab or Bendamustine plus Rituximab (NCT00868413), Dabrafenib plus Trametinib (NCT01989585), Venetoclax plus Ibrutinib and Rituximab (NCT05864742), Abiraterone acetate with or without Hydroxychloroquine (NCT01828476), and Bendamustine plus Rituximab (NCT01423539), were terminated with no results published, ongoing or were withdrawn.

Obatoclax mesylate, also known as GX15-070, is an inhibitor of BCL-2, BCL-xL, BCL-w, and MCL-1. This drug has also been tested in multiple clinical contexts. Phase I studies in hematologic malignancies and solid tumors demonstrated limited efficacy but acceptable safety, characterized mainly by neurological and psychiatric AEs, including somnolence and dizziness [342,343,344]. In AML, a few patients achieved stable disease while in myelodysplastic syndromes, no objective response was observed [345,346]. In myelofibrosis, hematologic improvement was observed in only one patient, whereas in Hodgkin’s lymphoma, the insufficient clinical responses led to a decision against further enrollment [347,348]. Other trials exploring Obatoclax mesylate in hematologic malignancies (NCT00438178) and systemic mastocytosis (NCT00918931) were completed with no published results.

Combination strategies with Obatoclax improved outcomes in certain settings. When added to Carboplatin/Etoposide in extensive-stage SCLC, it increased objective response rates to 62% vs. 53% for chemotherapy alone, without introducing unexpected toxicity [349,350]. In CLL, combination with Fludarabine and Rituximab produced complete and partial responses; however, neuropsychiatric effects were frequent [351]. In solid tumors and relapsed SCLC, the association with Topotecan achieved stable disease or partial responses, with hematologic and neurologic toxicity as primary limitations [352,353]. The combination with Bortezomib in mantle cell lymphoma yielded complete or partial responses in some patients, while association with Docetaxel in NSCLC achieved partial and stable responses, with frequent grade 3/4 neutropenia [354,355]. Other trials combining Obatoclax with Bortezomib (NCT00538187, NCT00719901), Vincristine/Doxorubicin/Dexrazoxane (NCT00933985), Rituximab with or without Bendamustine (NCT01238146, NCT00427856), and Carboplatin plus Etoposide (NCT01563601) were withdrawn, or completed without published results.

AT-101, also known as oral gossypol, is an orally bioavailable pan-BCL-2 inhibitor with activity against BCL-2 and BCL-xL that has also undergone extensive evaluation. Administration of AT-101 to refractory metastatic breast cancer patients led to a minor response and two patients achieving stable disease. Two out of the three patients receiving 50 mg/day had grade III dermatologic toxicity which was dose limiting. The MTD for this drug was 40 mg/day [356].

In castration-resistant prostate cancer, AT-101 demonstrated limited activity accompanied by gastrointestinal AEs, while in castration-sensitive metastatic prostate cancer, nearly one-third of patients achieved undetectable PSA levels [357,358]. Studies in glioblastoma multiforme yielded mostly stable disease, and activity was minimal in SCLC and adrenocortical carcinoma [359,360,361]. Trials in B-cell non-Hodgkin’s lymphoma (NCT05338931) remain ongoing, whereas one in relapsed or refractory B-cell malignancies (NCT00275431) was completed with no published results.

Combination regimens have shown comparatively better outcomes. AT-101 with Paclitaxel/Carboplatin produced objective responses in advanced solid tumors, and with Cisplatin/Etoposide achieved partial responses in approximately 15% of patients [362,363]. In gastroesophageal carcinoma, AT-101 combined with Docetaxel, 5-FU, and radiotherapy yielded high complete response rates [364]. In relapsed/refractory SCLC, combination with Topotecan induced partial and stable responses despite hematologic toxicity [365]. In head and neck cancer, AT-101 plus Docetaxel produced partial and stable responses, with lymphopenia as the main grade 3/4 AE [366]. In NSCLC, combinations with Docetaxel alone or Docetaxel plus Cisplatin resulted in disease stabilization in most patients, with neutropenia and anemia as the most common grade ≥ 3 AEs [367,368]. In laryngeal cancer, AT-101 with Docetaxel and platinum agents achieved partial responses in over half of treated patients [369]. The administration of AT-101, Docetaxel, and Prednisone in metastatic castration-resistant prostate cancer resulted in a median OS of 18.1 months; however, significant myelosuppression was observed [370]. A trial combining AT-101 with Erlotinib in NSCLC harboring EGFR mutations (NCT00988169) showed limited activity. Additionally, studies testing AT-101 with Temozolomide with or without radiotherapy (NCT00390403), Erlotinib (NCT00934076), Lenalidomide (NCT01003769 [296]), and Rituximab (NCT00286780, NCT00440388) were withdrawn or terminated with no published results.

Pelcitoclax, also known as APG-1252, is a dual BCL-2 and BCL-xL inhibitor that is being explored in several clinical trials. The results of the first clinical trial exploring Pelcitoclax in locally advanced or metastatic solid tumors led to three partial responses, one in SCLC, one in ovarian cancer, and another in a patient with neuroendocrine prostate cancer. Moreover, 11 patients achieved stable disease, leading to an overall disease control rate (DCR) of 30.4%. The recommended dose for further trials was a weekly dose of 240 mg of Pelcitoclax which in this trial led to a DCR of 50%. The regime was also found to be tolerable with the most common AEs being transaminase elevations and thrombocytopenia [371]. There are currently three active trials investigating Pelcitoclax: its combination with Cobimetinib in recurrent ovarian and endometrial cancers (NCT05691504), with Osimertinib in EGFR-TKI–resistant NSCLC (NCT04001777), and its use alone or with Chidamide in relapsed or refractory non-Hodgkin lymphoma (NCT05186012). In contrast, three monotherapy trials in SCLC or advanced solid tumors (NCT03387332), advanced neuroendocrine tumors (NCT04893759), and myelofibrosis after prior therapy (NCT04354727), as well as one evaluating Pelcitoclax plus Paclitaxel in relapsed/refractory SCLC (NCT04210037), were terminated or withdrawn without any published results.

More recently, AZD0466, a novel dual BCL-2/BCL-xL inhibitor which consists of a drug conjugate of AZD4320 and a DEP^®^ G5 poly-L-lysine dendrimer, entered early-phase clinical development. Trials in advanced hematologic or solid tumors (NCT04214093) were initiated, as well as studies evaluating AZD0466 combined with Voriconazole (NCT04865419) and with other anticancer agents in non-Hodgkin lymphoma (NCT05205161). One clinical trial was terminated with no published results, and the other two were terminated based on benefit–risk profile assessment.

Besides the limited efficacy observed with dual BCL-2 and BCL-xL inhibition as monotherapy, toxicity and the possibility of cancer cells to develop resistance are other major concerns for this type of inhibitors. Navitoclax, for example, is associated with neutropenia and dose-limiting thrombocytopenia since neutrophil progenitors and platelets are dependent on BCL-2 and BCL-xL, respectively, for their survival [372,373]. To mitigate thrombocytopenia, AZD4320 was developed; however, it exhibited dose-limiting cardiovascular toxicity in preclinical studies [372,374]. The drug-dendrimer conjugate AZD0466 was subsequently designed to overcome this limitation and showed comparable efficacy to AZD4320 with reduced toxicity, but clinical trials evaluating AZD0466 were terminated without published results [374].

Moreover, cancer cells can acquire resistance to BCL-2 and BCL-xL inhibitors through compensatory survival pathways. For instance, increased expression of MCL-1 and BFL-1 was found to be associated with resistance to ABT-737, a dual BCL-2/BCL-xL inhibitor [375]. In lymphoid and leukemic cells, Navitoclax resistance arises from high MCL-1 expression, while cells with high BCL-2 and NOXA mRNA levels show higher sensitivity to this drug [376].

Furthermore, BIM has been shown to interact with BCL-2 and BCL-xL through both its BH3 domain and C-terminal region. When both regions are connected to BCL-2 or BCL-xL, a mechanism known as double-bolt locking, displacement of these proteins is hindered, leading to resistance to Navitoclax and potentially to other BH3 mimetics [377].

Despite these limitations, clinical findings collectively indicate that these agents can enhance apoptotic signaling and overcome tumor cells’ resistance to several drugs [227,233,234,235,378]. Thus, not only could the targeting of BCL-2 pro-survival family members potentially promote cancer cells death through increased apoptotic signaling, but it also could potentially overcome resistance and increase the therapeutic potential of other drugs. Nevertheless, continued development of novel BCL-2 and BCL-xL–targeting strategies is essential to try and minimize toxicity, circumvent resistance mechanisms, and fully explore their therapeutic potential, particularly in combination regimens.

**Table 5:** Clinical trials exploring BCL-2 and BCL-xL inhibitors in the treatment of cancer.

Drug	Disease	Intervention	Phase	Results	NCT/References
Navitoclax	Lymphomas	Navitoclax	Phase I	Complete with no published results	NCT00743028
	Lymphoid malignancies and solid tumors	Navitoclax	Phase I	Complete with no published results	NCT00982566
	Lymphoid malignancies	Navitoclax	Phase I	Of the 46 patients analyzed, 10 showed partial response. The most common non-hematologic grade 3/4 AEs was pneumonia (11%).	NCT00406809 [323]
	SCLC and other solid tumors	Navitoclax	Phase I	Of the 38 patients analyzed, 8 showed stable disease and 1 a partial response. Serious AEs were reported in 3 patients (fatal respiratory failure, left ventricular systolic dysfunction and asymptomatic lipase elevation).	[324]
	Relapsed or refractory CLL	Navitoclax	Phase I	Among 29 patients, 7 achieved stable disease and 9 had a partial response. The MTD with intermittent dosing was estimated at 200 mg/day. Serious adverse events included grade ≥ 3 thrombocytopenia (*n* = 8), neutropenia (*n* = 8), tumor lysis syndrome (*n* = 1), progressive multifocal leukoencephalopathy (*n* = 1), and myocardial infarction (*n* = 1).	NCT00481091 [325]
	Relapsed or refractory high-risk myelodysplastic syndrome	Navitoclax	Phase Ib/II	Active, not recruiting	NCT05564650
	CLL	Navitoclax	Phase II	Complete with no published results	NCT01557777
	Relapsed or refractory lymphoid malignancies	Navitoclax	Phase IIa	All patients discontinued Navitoclax due to AEs and radiologic progressive disease.	NCT00406809 [326]
	Heavily pretreated recurrent epithelial ovarian cancer	Navitoclax	Phase II	Of the 46 patients analyzed, 15 showed stable disease and 1 a partial response. The most common grade 3/4 AEs was thrombocytopenia (26%).	NCT02591095 [328]
	Relapsed SCLC	Navitoclax	Phase II	Of the 39 patients analyzed, 9 showed stable disease and 1 a partial response. The most common AEs was thrombocytopenia (41%).	NCT00445198 [327]
	Relapsed or refractory B-cell CLL	Navitoclax	Phase IIb	Withdrawn since the sponsor decided to not proceed with the study	NCT00918450
	Lymphoma and solid tumors	Navitoclax with Ketoconazole	Phase I	Complete with no published results	NCT01021358
	SCLC	Navitoclax plus Etoposide and Cisplatin	Phase I	Complete with no published results	NCT00878449
	High grade serous epithelial ovarian cancer and TNBC	Navitoclax and Olaparib	Phase I	Active, not recruiting	NCT05358639
	Lymphoma and solid tumors	Navitoclax with Rifampin	Phase I	Complete with no published results	NCT01121133
	Relapsed/refractory AML	Navitoclax and Venetoclax with Cladribine-based salvage therapy	Phase I	Withdrawn since sponsor withdrew support	NCT06007911
	Advanced myeloid neoplasms	Navitoclax, Venetoclax and Decitabine	Phase I	Active, not recruiting	NCT05455294
	Relapsed/refractory AML	Navitoclax in combination with Venetoclax and Decitabine	Phase Ib	Active, not recruiting	NCT05222984
	Pediatric patients with relapsed or refractory hematological malignancies	Navitoclax in combination with Venetoclax and Decitabine	Phase I/II	Withdrawn since company stopped development and production of one of the investigational medicinal products.	NCT05740449
	Patients with myeloproliferative neoplasms	Navitoclax alone or in combination with Ruxolitinib	Phase I	Active, not recruiting	NCT04041050
	Myelofibrosis	Navitoclax, Ruxolitinib and Mivebresib vs. Mivebresib alone	Phase Ib	Terminated with no published results	NCT04480086
	Myelofibrosis	Navitoclax, Ruxolitinib and ABBV-744 vs. ABBV-744 alone	Phase Ib	Active, not recruiting	NCT04454658
	Myelofibrosis	Navitoclax with Ruxolitinib	Phase II	23–31% of the patients achieved a ≥35% spleen volume reduction and 24–30% a ≥50% total symptom score reduction. The most common AEs was reversible thrombocytopenia without clinically significant bleeding (88%).	NCT03222609 [338,379,380]
	Relapsed/refractory myelofibrosis	Navitoclax with Ruxolitinib	Phase III	Active, not recruiting	NCT04472598 NCT04468984
	Advanced solid tumors	Navitoclax and Vistusertib	Phase I	Treatment was well tolerated at dose level 1 (Navitoclax 150 mg once daily plus Vistusertib 35 mg twice daily). A serious AE, grade 3 serum aminotransferase elevation, occurred in 2 patients at dose level 2.	NCT03366103 [336]
	Relapsed or refractory solid tumors	Navitoclax and Sorafenib	Phase I	The MTD was Navitoclax 150 mg daily plus Sorafenib 400 mg twice daily. Stable disease was observed in 6 patients, with no partial or complete responses. The most common grade 3 toxicity was thrombocytopenia (*n* = 5).	NCT01364051 NCT02143401 [337]
	Solid tumors	Navitoclax combined with Gemcitabine	Phase I	Of the 46 patients analyzed, 21 showed stable disease. No complete or partial response was observed. The MTD was Navitoclax 325 mg plus Gemcitabine 1000 mg/m^2^. The most common AEs were thrombocytopenia 58.7%), nausea (58.7%), and fatigue (56.5%).	NCT00887757 [329]
	Advanced solid tumors	Navitoclax in combination with Erlotinib	Phase I	Among 11 patients, 3 showed stable disease with no complete or partial responses. The MTD was not reached. The most common grade ≥ 3 AEs were diarrhea (36%), syncope (18%), and thrombocytopenia (9%).	NCT01009073 [332]
	Advanced solid tumors	Navitoclax with Irinotecan	Phase I	Among 31 patients, 2 had partial responses. The MTD in a once-weekly regimen group was Navitoclax 150 mg plus Irinotecan 75 mg/m^2^. Grade ≥ 3 AEs occurred in 77.4% of patients, mostly diarrhea (35.5%).	NCT01009073 [334]
	Relapsed or refractory acute lymphoblastic leukemia and lymphoblastic lymphoma	Navitoclax and Venetoclax combined with chemotherapy	Phase I	Among 47 patients, 3 showed complete response, 8 a partial response, and 8 showed stable disease. The most common grade 3/4 AEs were febrile neutropenia (46.8%), neutropenia (38.3%), and thrombocytopenia (25.5%).	NCT03181126 [341]
	Advanced solid tumors	Navitoclax plus Docetaxel	Phase I	Of the 41 patients analyzed, 4 showed a partial response. The MTD was Navitoclax 150 mg days 1–5 every 21 days combined with Docetaxel 75 mg/m^2^ day 1. The most common AEs included thrombocytopenia (63%), fatigue (61%), nausea (59%) and neutropenia (51%).	NCT00888108 [330]
	Solid tumors	Navitoclax plus Carboplatin and Paclitaxel	Phase I	Among 19 patients, 1 showed a partial response. The study was discontinued due to significant hematological and non-hematological toxicities.	NCT00891605 [333]
	Relapsed or refractory chronic lymphocytic leukemia	Navitoclax plus Fludarabine, Cyclophosphamide and Rituximab or Bendamustine and Rituximab	Phase I	Complete with no published results	NCT00868413
	Relapsed or refractory CD20+ lymphoid malignancies	Navitoclax in combination with Rituximab	Phase I	Among 29 patients, 5 showed complete responses and 5 showed partial responses. The MTD of Navitoclax was 250 mg/day. Grade 4 thrombocytopenia occurred in 17% of patients, and common adverse events included mild diarrhea (79%) and nausea (72%).	NCT00788684 [339]
	B-cell chronic lymphocytic leukemia	Navitoclax with Rituximab	Phase II	Of the 78 patients analyzed, 2 showed complete responses, 47 showed partial responses, and 25 showed stable disease. Serious AEs were reported and included neutropenia (37.2%), and thrombocytopenia (25.6%).	NCT01087151 [340]
	EGFR-mutant NSCLC	Navitoclax plus Osimertinib	Phase Ib	Among 27 patients, 2 showed partial responses, and 12 showed stable disease. The recommended phase 2 dose was Osimertinib 80 mg plus Navitoclax 150 mg daily. The most common AEs were thrombocytopenia (37%), lymphopenia (37%), fatigue (22%), and nausea (22%).	NCT02520778 [335]
	Pediatric patients with relapsed or refractory acute lymphoblastic leukemia or lymphoblastic lymphoma	Navitoclax and Venetoclax	-	No results published	NCT05215405
	Relapsed acute lymphoblastic leukemia	Navitoclax and Venetoclax	Phase I/II	Active not recruiting	NCT05192889
	High-risk patients with T-cell acute lymphoblastic leukemia	Navitoclax and Venetoclax	Phase Ib/II	Not yet recruiting	NCT05054465
	KRAS or NRAS mutant advanced solid tumors	Navitoclax and Trametinib	Phase I/II	Among 91 patients, 8 showed partial responses. The recommended phase 2 dose was Trametinib 2 mg daily (days 1–14) plus Navitoclax 250 mg daily (days 1–28). The most common AEs included diarrhea (72.9%), decreased platelet count (70.6%) and increased AST (68.2%).	NCT02079740 [331]
	BRAF mutant melanoma and other solid tumors	Navitoclax with Dabrafenib and Trametinib	Phase I/II	Active not recruiting	NCT01989585
	Relapsed/refractory mantle cell lymphoma	Navitoclax plus Venetoclax, Ibrutinib and Rituximab vs. Venetoclax, Ibrutinib and Rituximab	Phase II	Active not recruiting	NCT05864742
	Progressive metastatic castrate refractory prostate cancer	Navitoclax and Abiraterone acetate with or without Hydroxychloroquine	Phase II	Terminated due to investigator leaving the organization	NCT01828476
	Relapsed DLBCL	Navitoclax plus Bendamustine and Rituximab	Phase II	Terminated due to non-safety related reasons	NCT01423539
Obatoclax Mesylate	Hematological malignancies	Obatoclax mesylate	Phase I	Completed with no published results	NCT00438178
	Advanced hematologic malignancies	Obatoclax	Phase I	Of the 44 patients analyzed, 1 showed a complete response and 3 showed hematologic improvement. Obatoclax mesylate was well tolerated with no dose-limiting toxicities. The most common AEs were somnolence (43%), dizziness (38%), and fatigue (36%).	[342]
	Advanced CLL	Obatoclax	Phase I	Of the 26 patients analyzed, 1 showed a partial response. The MTD was 28 mg/m^2^ over 3 h every 3 weeks. The most common grade ≥ 3 AEs were somnolence, ataxia, and confusion.	NCT00600964 [343]
	Advanced solid tumors or lymphoma	Obatoclax	Phase I	Of the 35 patients analyzed, 1 showed a partial response and 1 showed stable disease. The MTD was 1.25 mg/m^2^ for the 1 h infusion and 20 mg/m^2^ for the 3 h infusion. The most common AEs were somnolence (91%), dizziness (60%), and euphoric mood (57%).	[344]
	Previously untreated AML	Obatoclax mesylate	Phase I/II	Among 18 patients, 4 had stable disease, with no complete responses. The MTD was 20 mg/day administered over 3 h for 3 consecutive days. The most common AEs were neurologic (77.8%) or psychiatric (88.9%).	NCT00684918 [345]
	Systemic mastocytosis	Obatoclax	Phase II	No reported responses 3 months after treatment	NCT00918931
	Myelofibrosis	Obatoclax mesylate	Phase II	Among 22 patients, 1 showed clinical improvement in hemoglobin and platelets, with no complete or partial responses. The most common grade 3/4 AEs were fatigue (9%), dyspnea (9%), and febrile neutropenia (9%).	NCT00360035 [347]
	Hodgkin’s lymphoma	Obatoclax	Phase II	Among 13 patients, 5 had stable disease, with no objective responses. Further enrollment was not pursued due to limited clinical activity.	NCT00359892 [348]
	Patients with myelodysplastic syndromes with anemia or thrombocytopenia	Obatoclax	Phase II	Among 24 patients, 17 had stable disease, with no complete or partial responses. The most common grade 3/4 AEs were anemia (21%), thrombocytopenia (13%), and pneumonia (13%).	NCT00413114 [346]
	Aggressive relapsed or recurrent non-Hodgkin lymphoma	Obatoclax and Bortezomib	Phase I	Terminated with no results	NCT00538187
	Relapsed or refractory solid tumors, lymphoma, or leukemia	Obatoclax mesylate, Vincristine sulfate, Doxorubicin hydrochloride and Dexrazoxane hydrochloride	Phase I	Terminated with no results	NCT00933985
	Extensive-stage SCLC	Obatoclax in combination with Carboplatin and Etoposide	Phase I	Among 25 patients, 17 had complete or partial responses. The MTD of Obatoclax was 30 mg/day for the 3 h infusion. The most common AEs included neutropenia (96%), thrombocytopenia (76%), and anemia (72%).	[349]
	Extensive-stage SCLC	Obatoclax mesylate with Carboplatin and Etoposide vs. Carboplatin and Etoposide	Phase II	Among 155 patients, the objective response rate was 62% with Carboplatin/Etoposide plus Obatoclax and 53% with Carboplatin/Etoposide alone. Clinical benefit was 81% vs. 68%. Common grade 3/4 adverse events included neutropenia (46.5%), anemia (21%), and thrombocytopenia (16.5%).	NCT00682981 [350]
	Relapsed CLL	Obatoclax in combination with Fludarabine and Rituximab	Phase I	Among 13 patients, 2 had complete responses and 9 had partial responses. No MTD was reached. Common adverse events included dizziness (46%), euphoria (46%), and ataxia (38%).	NCT00612612 [351]
	Solid tumor malignancies	Obatoclax combined with Topotecan	Phase I	Of the 14 patients analyzed, 2 showed partial responses and 4 showed stable disease. The MTD of Obatoclax was 20 mg/m^2^. The most common grade 3/4 AEs were anemia (21.4%), and thrombocytopenia (14.3%).	[352]
	Relapsed SCLC	Obatoclax combined with Topotecan	Phase II	Of the 9 patients analyzed, 5 showed stable disease. No partial or complete responses were observed. The MTD of Obatoclax was 20 mg/m^2^. Common grade 3/4 AEs included thrombocytopenia (22%), anemia (11%), neutropenia (11%), and ataxia (11%).	NCT00521144 [353]
	Relapsed or refractory non-Hodgkin lymphoma	Obatoclax mesylate, Rituximab, and Bendamustine hydrochloride	Phase I/II	Withdrawn due to no patients accrued	NCT01238146
	Relapsed or refractory multiple myeloma	Obatoclax plus Bortezomib	Phase I/II	Trial termination was attributed to insufficient enrollment and drug supply	NCT00719901
	Relapsed or refractory mantle cell lymphoma	Obatoclax mesylate plus Bortezomib	Phase I/II	Of the 13 patients analyzed, 3 showed complete responses, 1 showed a partial response, and 6 showed stable disease. Grade 3/4 AEs included thrombocytopenia (21%), anemia (13%), and fatigue (13%).	NCT00407303 [354]
	Relapsed non–small-cell lung cancer	Obatoclax in combination with Docetaxel	Phase I/II	Of the 32 patients analyzed, 3 showed partial responses and 2 showed stable disease. The MTD was not reached. The most common grade 3/4 AEs included neutropenia (31%), febrile neutropenia (16%), and dyspnea (19%).	NCT00405951 [355]
	Previously untreated follicular lymphoma	Obatoclax mesylate with or without Rituximab	Phase II	Among 13 patients, 3 had complete responses, 1 had a partial response, and 6 had stable disease. Grade 3/4 adverse events included thrombocytopenia (21%), anemia (13%), and fatigue (13%).	NCT00427856
	Chemotherapy-naive patients with extensive-stage small cell lung cancer	Obatoclax mesylate combined with Carboplatin and Etoposide vs. Carboplatin and Etoposide	Phase III	Withdrawn due to business decision	NCT01563601
AT-101	Relapsed or refractory B-cell non-Hodgkin’s lymphoma	AT-101	Phase I/II	Currently recruiting	NCT05338931
	Refractory metastatic breast cancer	AT-101	Phase I/II	Grade III dose limiting dermatologic AEs were observed in two patients receiving 50 mg/day of AT-101. Stable disease was reported for 2 of the 20 patients analyzed in this study. A minor response was also reported. Moreover, the MTD was achieved (40 mg/day).	[356]
	Castrate-resistant prostate cancer	AT-101	Phase I/II	Of the 23 patients analyzed, 2 showed stable disease. Grade 3 small intestinal obstruction occurred in 21.7% of patients, while common AEs included diarrhea (43.5%), fatigue (34.8%), nausea (21.7%), and anorexia (21.7%).	NCT00286806 [357]
	Castration sensitive metastatic prostate cancer	AT-101	Phase II	Of the 55 patients analyzed, 17 (31%) achieved an undetectable PSA level (≤0.2 ng/mL). Serious AEs were reported in 22% of patients, and included sensory neuropathy (4%), and ileus (3.7%).	NCT00666666 [358]
	Relapsed or refractory B-cell malignancies	AT-101	Phase II	Complete with no published results	NCT00275431
	Newly diagnosed or recurrent glioblastoma multiforme	AT-101	Phase II	Among 71 patients, 1 had a partial response and 16 had stable disease. Grade 3/4 adverse events included gastrointestinal symptoms. All patients with recurrent glioblastoma multiforme died at the time of data analysis.	NCT00540722 NCT00390403 [359]
	Recurrent extensive stage SCLC	AT-101	Phase II	Of the 15 patients analyzed, 3 showed stable disease. No complete or partial responses were observed. Grade 3/4 AEs included anorexia (20%), fatigue (13.3%) and nausea (13.3%).	[360]
	Advanced adrenal cortical carcinoma	AT-101	Phase II	Of the 29 patients analyzed, 8 showed stable disease. No complete or partial responses were observed. The most commom grade 4 AEs were hypokalemia (10.3%), cardiac troponin elevation (3.4%), and lymphopenia (3.4%).	[361]
	Newly diagnosed glioblastoma multiforme	AT-101 and Temozolomide with or without radiotherapy	Phase I	Completed with no published results	NCT00390403
	Solid tumors	AT-101 with Paclitaxel and Carboplatin	Phase I	Of the 24 patients analyzed, 1 showed a complete response and 4 showed partial responses, and 8 showed stable disease. The MTD was not reached. Grade 3/4 AEs included neutropenia (21%), leukopenia (13%), anemia (4%), and thrombocytopenia (4%).	NCT00891072 [362]
	Advanced solid tumors	AT-101 with Cisplatin and Etoposide	Phase I	Of the 27 patients analyzed, 4 showed partial responses, and l0 demonstrated stable disease. The MTD of AT-101 was 40 mg orally twice daily on days 1–3, administered with Cisplatin 60 mg/m^2^ and Etoposide 120 mg/m^2^ on day 1 of a 21-day cycle, supported by Pegfilgrastim. The most common grade 3/4 AEs included neutropenia (59.3%), and leukopenia (44.4%).	[363]
	Advanced NSCLC	AT-101 with Erlotinib	Phase I	Withdrawn with no published results	NCT00934076
	Advanced NSCLC patients with EGFR activating mutations	AT-101 with Erlotinib	Phase II	Of the 5 patients analyzed, 3 showed stable disease and 1 a partial response. A serious adverse event was reported in 1 patient (hypotension)	NCT00988169
	Relapsed B-cell chronic lymphocytic leukemia	Lenalidomide and AT-101	Phase I/II	Of the 13 patients analyzed, 11 showed complete response. The most common AEs were gastrointestinal, including vomiting, anorexia, and odynophagia.	NCT01003769 [381]
	Gastroesophageal carcinoma	AT-101 with Docetaxel, 5-fluorouracil and radiotherapy	Phase I/II	Of the 36 patients analyzed, 3 showed partial responses and 15 showed stable disease. No complete response was observed. The most common AEs grade ≥ 3 were neutropenia (44.4%), thrombocytopenia (41.7%), and anemia (27.8%).	NCT00561197 [364]
	Relapsed and refractory SCLC	AT-101 with Topotecan	Phase I/II	Of the 13 patients analyzed, 11 showed complete response. The most common AEs were gastrointestinal, including vomiting, anorexia, and odynophagia.	NCT00397293 [365]
	CLL	AT-101 plus Rituximab	Phase II	Completed with no published results	NCT00286780
	Grade I-II follicular non-Hodgkin’s lymphoma	AT-101 combined with Rituximab	Phase II	Completed with no published results	NCT00440388
	Recurrent, locally advanced, or metastatic HNC	AT-101 in combination with Docetaxel	Phase II	Of the 11 evaluable patients, 1 showed partial responses and 6 showed stable disease. No complete response was observed. The most grade 3/4 AEs was lymphopenia (36.4%).	NCT01285635 [366]
	NSCLC	AT-101 plus Docetaxel	Phase II	Of the 53 patients analyzed, 2 showed partial response, and 22 showed stable disease. No complete responses were observed. The most common grade ≥ 3 AEs were neutropenia (9.4%), dyspnea (5.7%), and anemia (5.7%).	NCT00544960 [367]
	Advanced NSCLC with high expression of apurinic/apyrimidinic endonuclease 1	AT-101 combined with Docetaxel and Cisplatin	Phase II	Of the 31 patients analyzed, 3 showed partial response, and 23 showed stable disease. No complete responses were observed. The most common grade ≥ 3 AEs was anemia (3.2%).	NCT01977209 [368]
	Laryngeal cancer	AT-101 in combination with Docetaxel and Cisplatin or Carboplatin	Phase II	Of the 36 patients analyzed, 19 showed partial response. No complete responses were observed. The most common grade ≥ 3 AEs were neutropenia (9%), diarrhea (7%), and nausea (7%).	NCT01633541 [369]
	Metastatic castration-resistant prostate cancer	AT-101 plus Docetaxel and Prednisone Vs Docetaxel with Prednisone	Phase II	110 patients were analyzed. The median OS was 18.1 months. The most common grade 3/4 AEs were neutropenia (47.3%), leukopenia (24.5%), and lymphopenia (22.7%).	NCT00286793 NCT00571675 [370]
Pelcitoclax	SCLC or advanced solid tumors	Pelcitoclax	Phase I	Terminated with no published results	NCT03387332
	Locally advanced or metastatic solid tumors	Pelcitoclax	Phase I	The treatment was well tolerated with transaminase elevations and thrombocytopenia as most common AEs. The ORR and DCR were 6.5% and 30.4%, respectively.	NCT03080311 [371]
	Advanced neuroendocrine tumor	Pelcitoclax	Phase Ib	Terminated with no published results	NCT04893759
	Myelofibrosis that progressed after initial therapy	Pelcitoclax	Phase Ib/II	Withdrawn by sponsor decision	NCT04354727
	Recurrent ovarian and endometrial cancers	Pelcitoclax and Cobimetinib	Phase I	Currently recruiting	NCT05691504
	EGFR TKI resistant NSCLC	Pelcitoclax and Osimertinib	Phase Ib	Active, not recruiting	NCT04001777
	Relapsed or refractory non-Hodgkin lymphoma	Pelcitoclax or Pelcitoclax plus Chidamide	Phase Ib/II	Currently recruiting	NCT05186012
	Relapsed/refractory SCLC	Pelcitoclax and Paclitaxel	Phase Ib/II	Terminated with no published results	NCT04210037
AZD0466	Advanced hematologic or solid tumors	AZD0466	Phase I	Terminated with no published results	NCT04214093
	Advanced hematological malignancies	AZD0466 alone or combined with Voriconazole	Phase I/II	Terminated based on benefit-risk profile assessment	NCT04865419
	Advanced non-Hodgkin lymphoma	AZD0466 alone or combined with anticancer agents	Phase I/II	Terminated due to safety reasons	NCT05205161
LP-118	Advanced malignancies	LP-118	Phase I	Active, not recruiting	NCT05025358
	Relapsed or refractory hematological malignancies	LP-118	Phase I/Ib	Recruiting	NCT04771572
	Relapsed/refractory acute lymphoblastic leukemia or lymphoblastic lymphoma	LP-118, Ponatinib, Vincristine and Dexamethasone	Phase I/II	Recruiting	NCT06207123

## 7. Conclusions

BCL-2 and BCL-xL play a central role in apoptosis regulation, but their functions extend far beyond preventing cell death. They contribute to tumor progression, invasiveness, angiogenesis, chemotherapy resistance, and cellular metabolism, highlighting their multifaceted role in cancer biology. Although selective inhibitors, such as BH3 mimetics, show promise, challenges remain regarding efficacy and toxicity. Future research should continue to focus on developing combination therapies that target BCL-2 and BCL-xL alongside other oncogenic pathways, exploring context-specific inhibitors that minimize off-target effects, and identifying biomarkers to predict patient response. Additionally, deeper investigation into their non-apoptotic roles, including regulation of metabolism and mitochondrial dynamics, could uncover novel therapeutic opportunities and improve precision oncology approaches.

## Figures and Tables

**Table 3:** BCL-2 mRNA and protein expression according to UALCAN using The Cancer Genome Atlas (TCGA) and Clinical Proteomic Tumor Analysis Consortium (CPTAC) samples. Data were retrieved from UALCAN web resource (http://ualcan.path.uab.edu/, accessed on 14 January 2026), through the analysis of transcriptomic and proteomic data from TCGA and CPTAC, respectively, on 14 January 2026.

Organ	Cancer Type	mRNA Expression	Protein Expression
Bladder	Bladder urothelial carcinoma	Downregulated	No data
Brain and CNS	Glioblastoma multiforme	-	-
Breast	Breast cancer	No data	Downregulated
	Breast invasive carcinoma	Downregulated	No data
Cervix	Cervical squamous cell carcinoma	Downregulated	No data
Colon	Colon adenocarcinoma	Downregulated	No data
Endometrium	Uterine corpus endometrial carcinoma	Downregulated	-
Esophagus	Esophageal carcinoma	-	No data
Head and neck	Head and neck squamous cell carcinoma	-	-
Kidney	Clear cell renal cell carcinoma	Upregulated	-
	Kidney chromophobe	Upregulated	No data
	Renal papillary cell carcinoma	Upregulated	No data
Liver	Hepatocellular carcinoma	Upregulated	Downregulated
Lung	Lung adenocarcinoma	-	No data
	Lung squamous cell carcinoma	Downregulated	-
Pancreas	Pancreatic adenocarcinoma	-	No data
Prostate	Prostate adenocarcinoma	Downregulated	No data
Rectum	Rectum adenocarcinoma	Downregulated	No data
Stomach	Stomach adenocarcinoma	Downregulated	No data
Thymus	Thymoma	-	No data
Thyroid	Thyroid carcinoma	Downregulated	No data
Other	Cholangiocarcinoma	Upregulated	No data
	Pheochromocytoma and paraganglioma	-	No data
	Sarcoma	-	No data

**Table 4:** BCL-xL mRNA and protein expression according to UALCAN using TCGA and CPTAC samples. Data were retrieved from UALCAN web resource (http://ualcan.path.uab.edu/), through the analysis of transcriptomic and proteomic data from TCGA and CPTAC, respectively, on 14 January 2026.

Organ	Cancer Type	mRNA Expression	Protein Expression
Bladder	Bladder urothelial carcinoma	Upregulated	No data
Brain and CNS	Glioblastoma multiforme	-	-
Breast	Breast cancer	No data	-
	Breast invasive carcinoma	Upregulated	No data
Cervix	Cervical squamous cell carcinoma	Upregulated	No data
Colon	Colon adenocarcinoma	Upregulated	Upregulated
Endometrium	Uterine corpus endometrial carcinoma	Upregulated	Upregulated
Esophagus	Esophageal carcinoma	Upregulated	No data
Head and neck	Head and neck squamous cell carcinoma	Upregulated	Downregulated
Kidney	Clear cell renal cell carcinoma	-	Downregulated
	Kidney chromophobe	Upregulated	No data
	Renal papillary cell carcinoma	Upregulated	No data
Liver	Hepatocellular carcinoma	Upregulated	-
Lung	Lung adenocarcinoma	-	Upregulated
	Lung squamous cell carcinoma	Downregulated	Downregulated
Ovaries	Ovarian cancer	No data	Upregulated
Pancreas	Pancreatic adenocarcinoma	-	Upregulated
Prostate	Prostate adenocarcinoma	Upregulated	No data
Rectum	Rectum adenocarcinoma	Upregulated	No data
Stomach	Stomach adenocarcinoma	Upregulated	No data
Thymus	Thymoma	-	No data
Thyroid	Thyroid carcinoma	Upregulated	No data
Other	Cholangiocarcinoma	Upregulated	No data
	Pheochromocytoma and paraganglioma	-	No data
	Sarcoma	-	No data

## Data Availability

No new data were created or analyzed in this study. Data sharing is not applicable to this article.

## Abbreviations

The following abbreviations are used in this manuscript:

5-FU	5-fluorouracil
A1/BFL-1	BCL-2-related gene A1
AEs	Adverse events
AKT	Ak strain transforming
AML	Acute Myeloid Leukemia
APAF	Apoptotic Protease Activating Factor
ASF	Alternative splicing factor
ATP	Adenosine Triphosphate
BAD	BCL-2-associated death promoter
BAK	BCL-2 homologous antagonist/killer
BAX	BCL-2-associated X protein
BCL	B-cell lymphoma
BET	Bromodomain and extra-terminal
BFK	BCL-2 family kin
BH	BCL-2 Homology
BID	BH3-interacting domain death agonist
BIK	BCL-2-interacting killer
BIM	BCL-2-interacting mediator of cell death
BMF	BCL-2-modifying factor
BMPR2	Bone Morphogenetic Protein Receptor Type 2
BOK	BCL-2-related ovarian killer
BRAF	B-Raf proto-oncogene
Ca^2+^	Divalent calcium ion
CBP	CREB binding protein
CDK	Cyclin-dependent kinase
CeRNA	Competing Endogenous RNA
c-Jun	Cellular Jun
CK1ε	Casein kinase 1ε
CLL	Chronic lymphocytic leukemia
CML	Chronic myeloid leukemia
c-Myc	Cellular Myelocytomatosis oncogene
COX	Cyclooxygenase
CPTAC	Clinical Proteomic Tumor Analysis Consortium
CRC	Colorectal cancer
CRE	cAMP Response Element
CREB	cAMP Response Element-Binding protein
CXCR4	C-X-C chemokine receptor type 4
DCR	Disease control rate
DISC	Death-inducing signaling complex
DLBCL	Diffuse Large B-Cell Lymphoma
DNA	Deoxyribonucleic Acid
Drp1	Dynamin-related protein 1
DUSP4	Dual specificity phosphatase 4
EGFR	Epidermal growth factor receptor
ER	Endoplasmic reticulum
ERK	Extracellular signal-Regulated Kinase
FADD	Fas-associated protein with death domain
GABARAP	GABA type A Receptor–Associated Protein
GRIM19	Gene associated with Retinoid-IFN-induced Mortality 19
GTP	Guanosine Triphosphate
HCC	Hepatocellular carcinoma
HIF	Hypoxia-Inducible Factor
hnRNPs	Heterogeneous nuclear ribonucleoproteins
HNSCC	Head and Neck Squamous Cell Carcinoma
HRK	Harakiri
HSP	Heat Shock Protein
IDR	Intrinsically disordered region
IFN	Interferon
IL	Interleukin
IP3R	Inositol 1,4,5-trisphosphate receptor
JNK	c-Jun N-terminal kinase
LATS	Large tumor suppressor kinases
lncRNA	Long non-coding RNA
Lys	Lysine
MAPK	Mitogen-Activated Protein Kinase
MCL-1	Myeloid cell leukemia 1
MEK	Mitogen-activated protein kinase kinase
METTL14	Methyltransferase-like 14
miRNA	Micro Ribonucleic Acid
MITF	Microphthalmia-associated transcription factor
MMP	matrix metalloproteinase
MOMP	Mitochondrial outer membrane permeabilization
mRNA	Messenger Ribonucleic Acid
MST	Mammalian Sterile 20-like kinase
MTD	Maximum tolerated dose
MYC	Myelocytomatosis oncogene
NEK2	NIMA (Never In Mitosis Gene A)-related kinase 2
NF-κB	Nuclear Factor kappa-light-chain-enhancer of activated B cells
NFYA	Nuclear transcription factor Y α
NSCLC	Non-small cell lung cancer
OS	Overall survival
OSCC	Oral squamous cell carcinoma
PAR2	Protease-activated receptor 2
PARP	Poly (ADP-Ribose) Polymerase
PDTX	Patient-Derived Tumor Xenograft
PGAM5	Phosphoglycerate Mutase Family Member 5
PI3K	Phosphoinositide 3-Kinase
PINK1	PTEN-induced putative kinase 1
PKCα	Protein kinase Cα
PLK	Polo-like kinase
PP2A	Protein Phosphatase 2A
PTBP1	Polypyrimidine Tract Binding Protein 1
PTEN	Phosphatase and Tensin Homolog
PUMA	p53 upregulated modulator of apoptosis
RAS	Rat sarcoma
RASSF	Ras association domain family
RBM	RNA binding motif protein
ROS	Reactive oxygen species
RNAPII	RNA polymerase II
RNF	RING finger
RNPS1	RNA-binding protein with SR domain 1
SAC	Spindle assembly checkpoint
SAM68	SRC associated with mitosis, of 68 kDa
SAPK	Stress-Activated Protein Kinase
SCC	Squamous cell carcinoma
SCLC	Small cell lung cancer
Ser	Serine
SF3B1	Splicing factor 3b subunit 1
SOX9	SRY-box transcription factor 9
Sp1	Specificity Protein 1
SRPK	Serine/arginine protein kinase
SRSF	Serine/arginine-rich splicing factor
STAT	Signal Transducer and Activator of Transcription
TCGA	The Cancer Genome Atlas
TGF	Transforming Growth Factor
Thr	Threonine
TNBC	Triple-Negative Breast Cancer
TNF-R	Tumor necrosis factor receptor
TRA2β	Transformer 2β
TRAIL-R1/2	TNF-related apoptosis-inducing ligand receptor 1/2
UALCAN	University of ALabama at Birmingham CANcer data analysis Portal
Ub	Ubiquitination
VDAC1	Voltage-Dependent Anion Channel 1
VEGF	Vascular endothelial growth factor
VPS	Vacuolar Protein Sorting
uPA	Urokinase-type Plasminogen Activator
uPAR	Urokinase-type Plasminogen Activator Receptor
YAP	Yes-associated protein
